# Covering One Point Process with Another

**DOI:** 10.1007/s11009-025-10165-7

**Published:** 2025-04-29

**Authors:** Frankie Higgs, Mathew D. Penrose, Xiaochuan Yang

**Affiliations:** 1 Department of Mathematical Sciences, University of Bath, Bath, BA2 7AY UK; 2 Department of Mathematics, Brunel University London, Uxbridge, UB8 3PH UK

**Keywords:** Coverage threshold, Weak limit, Poisson point process, 60D05, 60F05, 60F15

## Abstract

Let $X_1,X_2, \ldots$ and $Y_1, Y_2, \ldots$ be i.i.d. random uniform points in a bounded domain $A \subset \mathbb {R}^2$ with smooth or polygonal boundary. Given $n,m,k \in \mathbb {N}$, define the *two-sample k-coverage threshold* $R_{n,m,k}$ to be the smallest *r* such that each point of $\{Y_1,\ldots ,Y_m\}$ is covered at least *k* times by the disks of radius *r* centred on $X_1,\ldots ,X_n$. We obtain the limiting distribution of $R_{n,m,k}$ as $n \rightarrow \infty$ with $m= m(n) \sim \tau n$ for some constant $\tau >0$, with *k* fixed. If *A* has unit area, then $n \pi R_{n,m(n),1}^2 - \log n$ is asymptotically Gumbel distributed with scale parameter 1 and location parameter $\log \tau$. For $k >2$, we find that $n \pi R_{n,m(n),k}^2 - \log n - (2k-3) \log \log n$ is asymptotically Gumbel with scale parameter 2 and a more complicated location parameter involving the perimeter of *A*; boundary effects dominate when $k >2$. For $k=2$ the limiting cdf is a two-component extreme value distribution with scale parameters 1 and 2. We also give analogous results for higher dimensions, where the boundary effects dominate for all *k*.

## Introduction

This paper is primarily concerned with the following *two-sample random coverage* problem. Given a specified compact region *B* in a *d*-dimensional Euclidean space, suppose *m* points $Y_j$ are placed randomly in *B*. What is the probability that these *m* points are fully covered by a union of Euclidean balls of radius *r* centred on *n* points $X_i$ placed independently uniformly at random in *B*, in the large-*n* limit with $m= m(n)$ becoming large and $r =r(n)$ becoming small in an appropriate manner?

In an alternative version of this question, the *X*-points are placed uniformly not in *B*, but in a larger region *A* with $B \subset A^o$ ($A^o$ denotes the interior of *A*). This version is simpler because boundary effects are avoided. We consider this version too.

We shall express our results in terms of the *two-sample coverage threshold* $R_{n,m}$, which we define to be the smallest radius of balls, centred on a set $\mathcal {X}_n$ of *n* independent uniform random points in *A*, required to cover all the points of a sample $\mathcal {Y}_m$ of *m* uniform random points in *B*. More generally, for $k \in \mathbb {N}$ the *two-sample k-coverage threshold* $R_{n,m,k}$ is the smallest radius required to cover $\mathcal {Y}_m$ *k* times. These thresholds are random variables, because the locations of the centres are random. We investigate their probabilistic behaviour as *n* and *m* become large.

A related question is to ask for coverage of the whole set *B*, not just of the point set $\mathcal {Y}_m$. We refer here to the smallest radius *r* such that *B* is contained in the union of the balls of radius *r* centred on points of *A*, as the *complete coverage threshold*. The asymptotic behaviour of this threshold has been addressed in Hall (1985) and Janson (1986) (for the case with $B \subset A^o$) and in Penrose (2023) (for the case with $B=A$). Clearly $R_{n,m}$ provides a lower bound for the complete coverage threshold.

Also related is the problem, when $m=n$ and $B=A$, of finding the *matching threshold*, that is, the minimum *r* such that a perfect bipartite matching of the samples $\mathcal {X}_n$ and $\mathcal {Y}_n$ exists with all edges of length at most *r*. This problem has been considered in Leighton and Shor (1989) and Shor et al. (1991), with applications to the theory of empirical measures. See e.g. García Trillos (2016) for recent application of results in Leighton and Shor (1989) and Shor et al. (1991) to clustering and classification problems in machine-learning algorithms.

Our problem is different since we allow the *X*-points to practice polygamy, and require all of the *Y*-points, but not necessarily all of the *X*-points, to be matched. Clearly $R_{n,n}$ is a lower bound for the matching threshold. This lower bound is asymptotically of a different order of magnitude than the matching threshold when $d=2$, but the same order of magnitude when $d \ge 3$. A slightly better lower bound is given by $\tilde{R}_{n,n}$, which we define to be the smallest *r* such that all *Y*-points are covered by *X*-points *and* all *X*-points are covered by *Y*-points. We expect that our methods can be used to show that $\lim _{n \rightarrow \infty } \mathbb {P}[\tilde{R}_{n,n} \le r_n] = \lim _{n \rightarrow \infty } \mathbb {P}[R_{n,n} \le r_n]^2$ for any sequence $(r_n)$ such that the limit exists, but proving this is beyond the scope of this paper. It is tempting to conjecture that the lower bound $\tilde{R}_{n,n}$ for the matching threshold might perhaps be asymptotically sharp as $n \rightarrow \infty$ in sufficiently high dimensions.

Another related problem is that of understanding the *bipartite connectivity threshold.* Given $\mathcal {X}_n, \mathcal {Y}_m$ and $r>0$, we can create a *bipartite random geometric graph* (BRGG) on vertex set $\mathcal {X}_n \cup \mathcal {Y}_m$ by drawing an edge between any pair of points $x \in \mathcal {X}_n, y \in \mathcal {Y}_m$ a distance at most *r* apart. The bipartite connectivity threshold is the smallest *r* such that this graph is connected, and the two-sample coverage threshold $R_{n,m}$ is a lower bound for the bipartite connectivity threshold. Two related thresholds are: the smallest *r* such that each point of $\mathcal {Y}_m$ is connected by a path in the BRGG to at least one other point of $\mathcal {Y}_m$, and the smallest *r* such that any two points of $\mathcal {Y}_m$ are connected by a path in the BRGG (but isolated points in $\mathcal {X}_n$ are allowed in both cases). Provided $m \ge 2$, these thresholds both lie between $R_{n,m}$ and the bipartite connectivity threshold, and have been studied in Iyer and Yogeshwaran (2012) and Penrose (2014).

Motivation for considering coverage problems comes from wireless communications technology (among other things); one may be interested in covering a region of land by mobile wireless transmitters (with locations modelled as the set of random points $X_i$). If interested in covering the whole region of land, one needs to consider the complete coverage threshold. In practice, however, it may be sufficient to cover not the whole region but a finite collection of receivers placed in that region (with locations modelled as the set of random points $Y_j$), and the two-sample coverage threshold addresses this problem. See Iyer and Yogeshwaran (2012) for further discussion of motivation from wireless communications.

See also Banerjee and Iyer (2013), which discusses a similar model where the $\mathcal {Y}$-sample represents a set of ‘sensors’ which cover space over short distances, and the $\mathcal {X}$-sample represents a set of ‘backbone nodes’ which communicate over longer distances. In Banerjee and Iyer (2013) the interest is in the volume of the region of space that is covered by sensors that are themselves covered by backbone nodes; a central limit theorem is derived for the volume of the complementary region. The quantity of interest to us here corresponds to the probability that all of the sensors are covered (at least *k* times) by backbone nodes.

We shall determine the limiting behaviour of $\mathbb {P}[R_{n,m(n),k} \le r_n]$ for any fixed *k*, any sequence $m(n)_{n \ge 1}$ of integers asymptotically proportional to *n*, and any sequence of numbers $(r_n)$ such that the limit exists, for the case where *B* is smoothly bounded (for general $d \ge 2$) or where *B* is a polygon (for $d =2$). We also obtain similar results for the Poissonized versions of this problem.

Our results show that when $d \ge 3$ the boundary effects dominate, i.e. the point of the $\mathcal {Y}$-sample furthest from its *k*-nearest neighbour in the $\mathcal {X}$-sample is likely to be near the boundary of *B*. When $d=2$, boundary effects are negligible for $k=1$ but dominate for $k \ge 3$. When $d=k=2$ the boundary and interior effects are of comparable importance; the point of the $\mathcal {Y}$-sample furthest from its second-nearest neighbour in the $\mathcal {X}$-sample has non-vanishing probability of being near the boundary of *B* but also non-vanishing probability of being in the interior.

In Section 6 we discuss the results of computer experiments, in which we sampled many independent copies of $R_{n,m(n),k}$ and plotted the estimated distributions of these radii (suitably transformed so that a weak law holds) alongside the limiting distributions we state in Section 2. These experiments motivated a refinement to our limit results, in which we explicitly included the leading-order error term, so that we can approximate the distribution of $R_{n,m(n),k}$ well for given finite *n*.

We work within the following mathematical framework. Let $d \in \mathbb {N}$. Let $A \subset \mathbb {R}^d$ be compact. Let $B \subset A$ be a specified Borel set (possibly the set *A* itself) with a nice boundary (in a sense to be made precise later on), and with volume $|B|>0$. Suppose on some probability space $(\mathbb {S},\mathcal {F},\mathbb {P})$ that $X_1,Y_1,X_2,Y_2,\ldots$ are independent random *d*-vectors with $X_i$ uniformly distributed over *A* and $Y_i$ uniformly distributed over *B* for each $i \in \mathbb {N}$. For $x \in \mathbb {R}^d$ and $r>0$ set $B_r(x) := B(x,r):= \{y \in \mathbb {R}^d:\Vert y-x\Vert \le r\}$ where $\Vert \cdot \Vert$ denotes the Euclidean norm. For $n \in \mathbb {N}$, let $\mathcal {X}_n:= \{X_1,\ldots ,X_n\}$ and let $\mathcal {Y}_{n,B}:= \{Y_1,\ldots ,Y_n\}$. Given also $m, k \in \mathbb {N}$, we define the *k*-coverage threshold $R_{n,m,k}$ by

1.1 $$\begin{aligned} R_{n,m,k}(B) : = \inf \left\{ r >0: \mathcal {X}_n (B(y,r)) \ge k ~~~~ \forall y \in \mathcal {Y}_{m,B} \right\} , ~~~ n,m,k \in \mathbb {N}, \end{aligned}$$

where for any point set $\mathcal {X}\subset \mathbb {R}^d$ and any $D \subset \mathbb {R}^d$ we write $\mathcal {X}(D)$ for the number of points of $\mathcal {X}$ in *D*, and we use the convention $\inf \{\}:= +\infty$. In particular $R_{n,m}(B): = R_{n,m,1}(B)$ is the two-sample coverage threshold. Observe that $R_{n,m}(B) = \inf \{ r >0: \mathcal {Y}_{m,B} \subset \cup _{i=1}^n B(X_i,r) \}$.

We are mainly interested in the case with $B=A$. In this case we write simply $\mathcal {Y}_m$, $R_{n,m,k}$ and $R_{n,m}$ for $\mathcal {Y}_{m,A}$, $R_{n,m,k}(A)$ and $R_{n,m}(A)$ respectively.

We are interested in the asymptotic behaviour of $R_{n,m}(B)$ for large *n*, *m*; in fact we take *m* to be asymptotically proportional to *n*. More generally, we consider $R_{n,m,k}(B)$ for fixed $k \in \mathbb {N}$.

We also consider analogous quantities denoted $R'_{t,u}(B)$ and $R'_{t,u,k}(B)$ respectively, defined similarly using Poisson samples of points. To define these formally, let $(Z_t,t\ge 0)$ be a unit rate Poisson counting process, independent of $(X_1,Y_1,X_2,Y_2,\ldots )$ and on the same probability space $(\mathbb {S},\mathcal {F},\mathbb {P})$ (so $Z_t$ is Poisson distributed with mean *t* for each $t >0$). Let $(Z'_t,t\ge 0)$ be a second unit rate Poisson counting process, independent of $(X_1,Y_1,X_2,Y_2,\ldots )$ and of $(Z_t,t \ge 0)$. The point process $\mathcal {P}_t:= \{X_1,\ldots ,X_{Z_t}\}$ is a Poisson point process in $\mathbb {R}^d$ with intensity measure $t \mu$, where we set $\mu$ to be the uniform distribution over *A* (see e.g. Last and Penrose 2018, Proposition 3.5). The point process $\mathcal {Q}_{t,B}:= \{Y_1,\ldots ,Y_{Z'_t}\}$ is a Poisson point process in $\mathbb {R}^d$ with intensity measure $t \nu$, where we set $\nu$ to be the uniform distribution over *B*. Then for $t,u \in (0,\infty ), k \in \mathbb {N}$ we define

1.2 $$\begin{aligned} R'_{t,u,k}(B) : = \inf \left\{ r >0: \mathcal {P}_t (B(y,r)) \ge k ~ \forall y \in \mathcal {Q}_{u,B} \right\} , \end{aligned}$$

with $R'_{t,u}:= R'_{t,u,1}$. When $B=A$ we write simply $\mathcal {Q}_{t}$, $R'_{t,u,k}$, $R'_{t,u}$, for $\mathcal {Q}_{t,A}$, $R'_{t,u,k}(A)$, $R'_{t,u,k}(A)$, respectively.

We mention some notation used throughout. For $D \subset \mathbb {R}^d$, let $\overline{D}$ denote the closure of *D*. Let |*D*| denote the Lebesgue measure (volume) of *D*, and $|\partial D|$ the perimeter of *D*, i.e. the $(d-1)$-dimensional Hausdorff measure of $\partial D$, when these are defined. Given $t >1$, we write $\log \log t$ for $\log (\log t)$. Let *o* denote the origin in $\mathbb {R}^d$.

Let $\theta _d$ denote the volume of a unit radius ball in $\mathbb {R}^d$. Set $f_0:= 1/|A|$.

If $t_0 \in (0,\infty )$ and *f*(*t*), *g*(*t*) are two functions, defined for all $t \ge t_0$ with $g(t) >0$ for all $t \ge t_0$, the notation $f(t)= O(g(t))$ as $t \rightarrow \infty$ means that $\limsup _{t \rightarrow \infty } (|f(t)|/g(t)) < \infty$, and the notation $f(t)= o(g(t))$ as $t \rightarrow \infty$ means that $\limsup _{t \rightarrow \infty } (|f(t)|/g(t)) =0.$ If also $f(t) >0$ for all $t \ge t_0$, we use notation $f(t)= \Theta (g(t))$ to mean that both $f(t)=O(g(t)$ and $g(t)= O(f(t))$.

## Statement of Results

Our results are concerned with weak convergence for $R_{n,m,k}(B)$ (defined at Eq. 1.1) as $n \rightarrow \infty$ with *k* fixed and *m* asymptotically proportional to *n*. We also give similar results for $R'_{t,\tau t,k}$, defined at Eq. 1.2, as $t \rightarrow \infty$ with $\tau >0$ also fixed. In all of these limiting results we are taking the variable *n* to be integer-valued and *t* to be real-valued.

Recall that our $\mathcal {X}$-sample is of points uniformly distributed over a compact region $A \subset \mathbb {R}^d$, and the $\mathcal {Y}$-sample is of points in *B*, where $B \subset A$ has a ‘nice’ boundary. We now make this assumption more precise. We always assume one of the following:

1. $d \ge 2$ and $B=A$ and *A* has a $C^{1,1}$ boundary and $\overline{A^o}=A$, or
2. $d = 2$ and $B=A$ and *A* is polygonal, or
3. $d \ge 2$ and $\overline{B} \subset A^o$, and *B* is Riemann measurable with $|B| >0$. (Recall that a compact set *B* is said to be Riemann measurable if $\partial B$ is Lebesgue-null.)

We say that *A* *has a* $C^{1,1}$ *boundary* if for each $x \in \partial A$ there exists a neighbourhood *U* of *x* and a real-valued function *f* that is defined on an open set in $\mathbb {R}^{d-1}$ and Lipschitz-continuously differentiable, such that $\partial A \cap U$, after a rotation, is the graph of the function *f*. The $C^{1,1}$ boundary condition is milder than the $C^2$ boundary condition that was imposed for analogous results on the complete coverage threshold in Penrose (2023). The extra condition $\overline{A^o} =A$ should also have been included in Penrose (2023) to rule out examples such as the union of a disk and a circle in $\mathbb {R}^2$.

For compact $A \subset \mathbb {R}^d$ satisfying A1 or A2, let |*A*| denote the volume (Lebesgue measure) of *A* and $|\partial A|$ the perimeter of *A*, i.e. the $(d-1)$-dimensional Hausdorff measure of $\partial A$, the topological boundary of *A*. Also define

2.1 $$\begin{aligned} \sigma _A := \frac{|\partial A|}{|A|^{1-1/d}}. \end{aligned}$$

Note that $\sigma _A$ is invariant under scaling of *A*, and is at least $d \theta _d^{1/d}$ by the isoperimetric inequality. Sometimes $\sigma _A^d$ is called the *isoperimetric ratio* of *A*.

Our first result concerns the case with $\overline{B} \subset A^o$. Recall that $f_0:=1/|A|$.

### Theorem 2.1

(Fluctuations of $R_{n,m,k}(B)$ when $\overline{B} \subset A^o$) Suppose A3 applies. Let $k \in \mathbb {N}$

and $\tau >0, \beta \in \mathbb {R}$. Let $m: \mathbb {N}\rightarrow \mathbb {N}$, and assume $\tau _n: = m(n) /n \rightarrow \tau$ as $n \rightarrow \infty$. Then as $n \rightarrow \infty$ we have

2.2 $$\begin{aligned}&\mathbb {P}[ n \theta _d f_0 R_{n,m(n),k}(B)^d - \log n - (k-1) \log \log n \le \beta ] \nonumber \\&~~~~ = \exp \left( - \frac{\tau _n e^{-\beta }(k-1)^2 \log \log n}{(k-1)!\log n} \right) e^{-(\tau _ne^{-\beta })/(k-1)!} + O((\log n)^{-1}). \end{aligned}$$

Also as $t \rightarrow \infty$ we have

2.3 $$\begin{aligned}&\mathbb {P}[ t \theta _d f_0 (R'_{t,\tau t,k}(B))^d - \log t - (k-1) \log \log t \le \beta ] \nonumber \\&~~~~ = \exp \left( - \frac{\tau e^{-\beta }(k-1)^2 \log \log t}{(k-1)!\log t} \right) e^{-(\tau e^{-\beta })/(k-1)!} + O((\log t)^{-1}). \end{aligned}$$

### Remark

1. Given $\xi \in \mathbb {R}$, $\theta \in (0,\infty )$, let ${\textsf{Gu}}_{\xi ,\theta }$ denote a Gumbel random variable with location parameter $\xi$ and scale parameter $\theta$, i.e. with cumulative distribution function (cdf) $F(x) = \exp (-e^{-(x-\xi )/\theta })$. Since the right hand side of Eq. 2.2 converges to $\exp (-(\tau e^{-\beta })/(k-1)!)$ as $n \rightarrow \infty$, it follows from Eq. 2.2 that as $n \rightarrow \infty$ we have the convergence in distribution:
  $$ n \theta _d f_0 R_{n,m(n),k}(B)^d - \log n - (k-1) \log \log n \overset{\mathcal {D}}{\longrightarrow }{\textsf{Gu}}_{\log (\tau /(k-1)!),1}. $$
  Similarly, as $t \rightarrow \infty$, by Eq. 2.3 we have
  $$ n \theta _d f_0 R'_{t,\tau t,k}(B)^d - \log t - (k-1) \log \log t \overset{\mathcal {D}}{\longrightarrow }{\textsf{Gu}}_{\log (\tau /(k-1)!),1}. $$
2. The $O((\log n)^{-1})$ term in Eq. 2.2 and the $O((\log t)^{-1})$ term in Eq. 2.3 come partly from an error bound of $O((\log t)^{1-d})$ in a Poisson approximation for the number of isolated points; see Lemma 4.1. If $d \ge 3$ the error bound in the Poisson approximation is of higher order, and hence we can give a more accurate approximation with an explicit $(\log n)^{-1}$ term (respectively, $(\log t)^{-1}$ term) included in the first exponential factor on the right, and an error of $O((\frac{\log \log n}{\log n})^2)$ in Eq. 2.2 (resp., of $O((\frac{\log \log t}{\log t})^2)$ in Eq. 2.3). See Eqs. 5.3 and 5.4 in the proof of Theorem 2.1 for details.

All of our remaining results are for the case $B=A$.

First we briefly discuss the case where *A* is the *d*-dimensional unit *torus* (and $B=A$). In this case, taking $f_0=1$, we can obtain exactly the same result as stated in Theorem 2.1, by the same proof. We note that a result along these lines (for $k=1$ only) has been provided previously (with a different proof) in (Iyer and Yogeshwaran 2012, Theorem 3.2), for $\tau$ large. Iyer and Yogeshwaran (2012) is more concerned with the threshold *r* such that each vertex of $\mathcal {Y}_m$ has a path to at least one other point of $\mathcal {Y}_m$ in the BRGG. In any event, the authors of Iyer and Yogeshwaran (2012) explicitly restrict attention to the torus, in their words, to ‘nullify some of the technical complications arising out of boundary effects’. In our next results, we embrace these technical complications.

We next give our main result for $d=2$, $k=1$.

### Theorem 2.2

(Fluctuations of $R_{n,m}$ in a planar region with boundary) Suppose $d=2$ and A1 or A2 holds. Set $f_0:= |A|^{-1}$. Let $\beta ,\tau \in \mathbb {R}$ with $\tau >0$. Suppose $m:\mathbb {N}\rightarrow \mathbb {N}$ with $\tau _n:= m(n)/n \rightarrow \tau$ as $n \rightarrow \infty$. Then as $n \rightarrow \infty$,

2.4 $$\begin{aligned} \mathbb {P}\left[ n \pi f_0 R_{n,m(n)}^2 - \log n \le \beta \right] = \exp \Big (- \frac{\tau _n \pi ^{1/2} \sigma _A e^{-\beta /2} }{ 2 (\log n)^{1/2}} \Big ) e^{ - \tau _n e^{- \beta } } + O((\log n)^{-1}) . \end{aligned}$$

Also, as $$t \rightarrow \infty $$,

2.5 $$\begin{aligned} \mathbb {P}\left[ t \pi f_0 (R'_{t,\tau t})^2 - \log t \le \beta \right] = \exp \Big (- \frac{\tau \pi ^{1/2} \sigma _A e^{-\beta /2} }{ 2 (\log t)^{1/2}} \Big ) \exp \left( - \tau e^{- \beta } \right) + O((\log t)^{-1}) . \end{aligned}$$

### Remark

It follows from Eq. 2.4 that $n \pi f_0 R_{n,m(n)}^2 -\log n \overset{\mathcal {D}}{\longrightarrow }{\textsf{Gu}}_{\log \tau ,1}$. Denoting the median of the distribution of any continuous random variable *Z* by $\mu (Z)$, we have $\mu (n\pi f_0 R_{n,m(n)}^2) = \log n +\mu ({\textsf{Gu}}_{\log \tau ,1}) + o(1)$. We can subtract the medians from both sides, and then we have $n \pi f_0 R_{n,m(n)}^2 - \mu (n \pi f_0 R_{n,m(n)}^2) \overset{\mathcal {D}}{\longrightarrow }{\textsf{Gu}}_{\log \log 2,1}$, where ${\textsf{Gu}}_{\log \log 2,1}$ is a Gumbel random variable with scale parameter 1 and median 0. The second row of Fig. 5 illustrates each of these two convergences in distribution. It is clearly visible that subtracting the median gives a much smaller discrepancy between the distribution of $n \pi f_0 R_{n,m(n)}^2 - \mu (n \pi f_0 R_{n,m(n)}^2)$ and its limit, suggesting that $\mu (n\pi f_0 R_{n,m(n)}^2)-\log n \rightarrow \mu ({\textsf{Gu}}_{\log \tau ,1})$ quite slowly. However, we estimated $\mu (n \pi f_0 R_{n,m(n)}^2)$ using the sample median of a large number of independent copies of $n \pi f_0 R_{n,m(n)}^2$. When applying estimates such as Eq. 2.4 to real data, a large number of samples may not be available, and we do not currently have an expression for $\mu (n\pi f_0 R_{n,m(n)}^2)-\log n - \mu ({\textsf{Gu}}_{\log \tau ,1})$.

Simulations with *A* taken to be a disk or square suggest that even for quite large values of *n*, with $m(n) = \lfloor \tau n \rfloor$ for some fixed $\tau$, the estimated cdf of $n \pi f_0 R_{n,m(n)}^2 - \log n$ from simulations does not match the limiting Gumbel cdf particularly well. This can be seen in the bottom-left plot of Fig. 5, where the estimated cdf (the blue curve) is not well-approximated by the limit (the black dashed curve). This is because the multiplicative correction factor of $\exp (-\tau _n (\pi ^{1/2}/2) \sigma _A e^{-\beta /2} (\log n)^{-1/2})$, which we see in Eq. 2.4, tends to 1 very slowly. (We have written it as a multiplicative correction to ensure that the right hand side is a genuine cdf in $\tau _n$ plus an $O((\log n)^{-1})$ error term.)

If instead we compare the cdf of $n \pi f_0 R_{n,m(n)}^2-{\log n}$ estimated by simulations with the corrected cdf $F(x) = \exp (- \frac{\pi \tau _n \sigma _Ae^{-x/2}}{{2}(\log n)^{1/2}}) \exp (-\tau _n e^{-x})$, illustrated as a red dotted line in the same part of Fig. 5, we get a much better match.

Next we give results for $d=2, k \ge 2$ and for $d \ge 3$. Given (*d*, *k*) we define the constant

2.6 $$\begin{aligned} c_{d,k} := \frac{\theta _d^{1-1/d} (1- 1/d)^{k-2+1/d}}{(k-1)! 2^{1-1/d} \theta _{d-1}}, \end{aligned}$$

### Theorem 2.3

Suppose A1 or A2 holds.

Let $\beta ,\tau \in \mathbb {R}$ with $\tau >0$. Suppose $m:\mathbb {N}\rightarrow \mathbb {N}$ with $\tau _n:= m(n)/n \rightarrow \tau$ as $n \rightarrow \infty$, and for $n \in \mathbb {N}$, $t >0$ let

$$\begin{aligned} u_n := \mathbb {P}[n \theta _d f_0 R_{n,m(n),k}^d - (2-2/d) \log n - (2k -4 +2/d) \log \log n) \le \beta ]; \\ u'_t := \mathbb {P}[t \theta _d f_0 (R'_{t,\tau t,k})^d - (2-2/d) \log t - (2k-4 + 2/d) \log \log t) \le \beta ]. \end{aligned}$$

If $(d,k) = (2,2)$ then, with $g_A(\beta ):=8e^{-\beta }+\pi ^{1/2}\sigma _A e^{-\beta /2}$, as $n \rightarrow \infty$,

2.7 $$\begin{aligned} u_n = \exp \Big ( - \frac{\tau _n g_A{(\beta )} \log \log n}{ 8 \log n} \Big ) \exp \left( - \tau _n \left( e^{-\beta } + \frac{\pi ^{1/2}\sigma _A e^{-\beta /2}}{4}\right) \right) + O \big ( \frac{1}{ \log n} \big ), \end{aligned}$$

and as $$t \rightarrow \infty $$,

2.8 $$\begin{aligned} u'_t = \exp \Big ( - \frac{\tau g_A{(\beta )} \log \log t}{ 8 \log t} \Big ) \exp \left( - \tau \left( e^{-\beta } + \frac{\pi ^{1/2} \sigma _A e^{-\beta /2}}{4}\right) \right) + O \big ( \frac{1}{ \log t} \big ). \end{aligned}$$

If $d=2, k \ge 3$ or if $d \ge 3$ then as $n \rightarrow \infty$,

2.9 $$\begin{aligned} u_n = \exp \Big ( - \frac{ c_{d,k} \tau _n \sigma _A e^{-\beta /2} (k-2+1/d)^2 \log \log n}{ (1-1/d) \log n} \Big ) \exp \left( - c_{d,k} \tau _n \sigma _A e^{-\beta /2} \right) \nonumber \\ + O\Big (\frac{1}{\log n}\Big ) \end{aligned}$$

and as $$t \rightarrow \infty $$,

2.10 $$\begin{aligned} u'_t = \exp \Big ( - \frac{ c_{d,k} \tau \sigma _A e^{-\beta /2} (k-2+1/d)^2 \log \log t}{ (1-1/d) \log t} \Big ) \exp \left( - c_{d,k} \tau \sigma _A e^{-\beta /2} \right) \nonumber \\ + O\Big (\frac{1}{\log t}\Big ). \end{aligned}$$

### Remark

1. It follows from Eqs. 2.9, 2.10 that when $d =2, k \ge 3$ or $d \ge 3$ we have as $n \rightarrow \infty$ that
  $$ n \theta _d f_0 R_{n,m(n),k}^d - (2-2/d) \log n - (2k-4+2/d) \log \log n \overset{\mathcal {D}}{\longrightarrow }{\textsf{Gu}}_{\log (c_{d,k} \tau \sigma _A),2}, $$
  along with a similar result for $R_{t,\tau t,k}$. On the other hand, when $d=2,k=2$ we have from Eq. 2.7 that
  $$ n \pi f_0 R_{n,m(n),2}^2 - \log n - \log \log n \overset{\mathcal {D}}{\longrightarrow }\max ({\textsf{Gu}}_{\log \tau ,1},{\textsf{Gu}}'_{\log (\tau \pi ^{1/2} \sigma _A/4),2}), $$
  where ${\textsf{Gu}}$ and ${\textsf{Gu}}'$ denote two independent Gumbel variables with the parameters shown. The distribution of the maximum of two independent Gumbel variables with different scale parameters is known as a *two-component extreme value* (TCEV) distribution in the hydrology literature (Rossi et al. 1984).
2. As in the case of Theorem 2.1, when $d \ge 3$ in Theorem 2.3 we could replace the $O((\log n)^{-1})$ remainder in Eq. 2.9 with an explicit $(\log n)^{-1}$ term and an $O((\frac{\log \log n}{\log n})^2)$ remainder, and likewise for the $O((\log t)^{-1})$ remainder in Eq. 2.10; see Eqs. 5.16 and 5.15 in the proof of Theorem 2.3 for details.

Comparing these results with the corresponding results for the complete coverage threshold (Hall 1985; Janson 1986; Penrose 2023), we find that the typical value of that threshold (raised to the power *d* and then multiplied by *n*) is greater than the typical value of our two-sample coverage threshold (transformed the same way) by a constant multiple of $\log \log n$. For example, our Theorem 2.1 has a coefficient of $k-1$ for $\log \log n$ while (Penrose 2023, Proposition 2.4) has a coefficient of *k*. When $d=2, k=1$, our Theorem 2.2 has a coefficient of zero for $\log \log n$ whereas (Penrose 2023, Theorem 2.2) has a coefficient of 1/2.

We shall prove our theorems using the following strategy. Fix $k \in \mathbb {N}$. Given $t,r >0$ define the random ‘vacant’ set

2.11 $$\begin{aligned} V_{t,r,k}:= \{x \in A: \mathcal {P}_t (B(x,r)) < k\}. \end{aligned}$$

Given $\gamma \in (0,\infty )$, suppose we can find $(r_t)_{t >0}$ such that $t \mathbb {E}[|V_{t,r_t,k} \cap B|]/|B| = \gamma$.

If we know $t |V_{t,r_t,k} \cap B|/|B| \approx \gamma$, then the distribution of $\mathcal {Q}_{\tau t,B} (V_{t,r_t,k})$ is approximately Poisson with mean $\tau \gamma$, and we use the Chen-Stein method to make this Poisson approximation quantitative, and hence show that $\mathbb {P}[R'_{t,\tau t,k} \le r_t]$ approximates to $e^{- \tau \gamma }$ for *t* large (see Lemma 4.1). By coupling binomial and Poisson point processes, we obtain a similar result for $\mathbb {P}[R_{n,m(n),k} \le r_n]$ (see Lemma 4.2).

Finally, we need to find nice limiting expression for $$r_t$$ as $$t \rightarrow \infty $$. By Fubini’s theorem $$\mathbb {E}[|V_{t,r_t,k} \cap B|]= \int _B p_t(x)dx$$, where we set $$p_t(x)= \mathbb {P}[x \in V_{t,r_t,k}]$$. Hence we need to take $$r_t \rightarrow 0$$. Under A3, for *t* large $$p_t(x) $$ is constant over $$x \in B$$ so finding a limiting expression for $$r_t$$ in that case is fairly straightforward.

Under Assumption A1 or A2, we need to deal with boundary effects since $p_t(x)$ is larger for *x* near the boundary of *A* than in the interior (or ‘bulk’) of *A*. In Lemma 3.7 we determine the asymptotic behaviour of the integral near a flat boundary; since the contribution of corners turns out to be negligible this enables us to handle the boundary contribution under A2.

Under Assumption A1, we need to deal with integrals of $p_t(x)$ over *x* near the curved boundary of *A*. We approximate $p_t(x)$ by a function depending only on $\,\textrm{dist}(x,\partial A) := \inf _{y \in \partial A} \Vert x-y\Vert$, and parameterize *x* by the nearest point in $\partial A$ and the distance from $\partial A$. In Proposition 3.8 we provide a useful estimate on the Jacobian arising from this parameterization. The upshot is that we can reduce the integral to a one-dimensional integral that can be dealt with using Lemma 3.7.

Alternatively it is possible to handle the curved boundary by adapting methodology of Penrose (2023), whereby one approximates *A* by a polytope $A_t$ with spacing that tends to zero more slowly than $r_t$. In an earlier version of this paper (v1 on ArXiv) this alternative method is carried out. However the method developed here, using Proposition 3.8, seems to provide a cleaner proof and is likely to be useful in other settings.

It turns out that $d=2,k=1$ is a special case because in this case only, the contribution of the bulk dominates the contribution of the boundary region to $\mathbb {E}[|V_{t,r_t,k}|]$. When $d=2,k=2$ both contributions are equally important, and in all other cases the boundary contribution dominates the contribution of the bulk. This is why the formula for the centring constant for $R'_{t,\tau t,k}$ or $R_{n,m,k}$ in terms of *d* and *k* is different for Theorem 2.2 than for Theorem 2.3 (the coefficient of $\log \log n$ being 0 rather than 1 in Theorem 2.2), and why in Theorem 2.3 the limiting distribution is TCEV for $d=k=2$ but is Gumbel for all other cases.

## Preparatory Results

We use the following notation from time to time. Given $r >0$, and $A \subset \mathbb {R}^d$, set $\partial A^{(r)} := A \cap \cup _{x \in \partial A} B_r(x)^o$. Aso set $A^{(-r)}:= A \setminus \partial A^{(r)}$.

Let $\pi : \mathbb {R}^d \rightarrow \mathbb {R}^{d-1}$ denote projection onto the first $d-1$ coordinates and let $e_d:= (0,\ldots ,0,1)$, the *d*th coordinate vector in $\mathbb {R}^d$. Let $x \cdot y$ denote the Euclidean inner product of vectors $x,y \in \mathbb {R}^d$. For $a \in [0,1]$, let

3.1 $$\begin{aligned} h(a) := |B_1(o) \cap ([0,a] \times \mathbb {R}^{d-1})|. \end{aligned}$$

We suppress the dependence of *h*(*a*) on on the dimension *d*.

Throughout this section we assume that $A \subset \mathbb {R}^d$ is bounded with a $C^{1,1}$ boundary, and that $A = \overline{A^\circ }$.

### Geometrical Lemmas

#### Definition 3.1

(Sphere condition) For $z \in \partial A$ let $\hat{n}_z$ be the unit normal to $\partial A$ at *z* pointing inside *A*.

Given $\tau \ge 0$, let us say $\tau$ satisfies the *sphere condition* for *A* if, for all $x \in \partial A$, we have $B(x+ \tau \hat{n}_x, \tau ) \subset A$ and $B(x -\tau \hat{n}_x, \tau ) \cap A = \{x\}$.

Let $\tau (A)$ denote the supremum of the set of all $\tau$ satisfying the sphere condition for *A*.

#### Lemma 3.2

(Sphere condition lemma) $\tau (A) >0$; that is, there exists a constant $\tau >0$ such that $\tau$ satisfies the sphere condition for *A*.

#### Proof

See (Lewicka and Peres 2020, Lemma 7). $\square$

#### Remark 3.3

(i) If $0< \tau < \tau '$ and $\tau '$ satisfies the sphere condition for *A*, then so does $\tau$.
(ii) If $x \in \mathbb {R}^d$ with $\,\textrm{dist}(x,\partial A) < \tau (A)$, then *x* has a unique closest point in $\partial A$.

Given small $$r >0 $$, and $$x \in \partial A^{(r)}$$, we are interested in estimating the volume of $$A \cap B(x,r)$$. Using the sphere condition we can approximate this volume with that of a certain ‘sliced ball’.

For $x\in A$ let $a(x): = \,\textrm{dist}(x,\partial A)$, the Euclidean distance from *x* to $\partial A$. For $x \in \partial A^{(r)}$, we shall approximate $| B_r(x) \cap A |$ by $(\frac{1}{2}\theta _d + h(a(x)/r))r^d$, the volume of the portion of $B_r(x)$ which lies on one side of the tangent plane to $\partial A$ at the closest point to *x* on $\partial A$.

#### Lemma 3.4

Suppose $0< r < \tau (A)$, and $x \in \partial A^{(r)}$. Then

3.2 $$\begin{aligned} \left| |B_r(x) \cap A| - ((\theta _d/2) + h(a(x)/r))r^d \right| \le \frac{2 \theta _{d-1} r^{d+1}}{\tau (A)}. \end{aligned}$$

#### Proof

Without loss of generality the closest point on the boundary to *x* is the origin *o* and $x = a e_d$ for $a = a(x) \in [0,r)$. Let $\mathbb {H}:= \{ y \in \mathbb {R}^{d}: y \cdot e_d \ge 0 \}$ the upper half-space, and note that $| B_r(x) \cap \mathbb {H}| = ((\theta _d/2) + h(a/r))r^d$, the volume we are using to approximate $|B_r(x) \cap A|$.

**Fig. 1:**
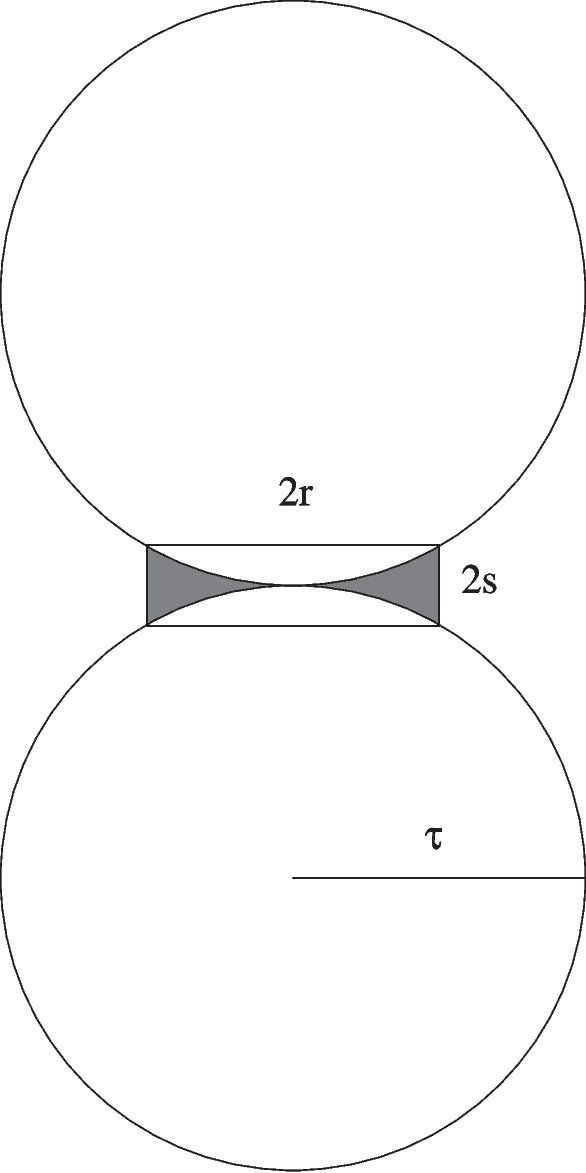
Illustration for proof of Lemma 3.4. The set $(B_r(x) \cap A) \triangle (B_r(x) \cap \mathbb {H})$ is contained in the shaded region

Let $\tau \in (r, \tau (A))$. Let $S:= B_\tau (\tau e_d)^o$ and $S':= B_\tau (-\tau e_d)^o$. Then the set $(B_r(x) \cap A) \triangle (B_r(x) \cap \mathbb {H})$ is contained in $\mathbb {R}^d \setminus (S \cup S')$. Therefore by some spherical geometry, it is contained in a cylinder *C* centred on *o* of radius *r* and height 2*s*, as illustrated in Fig. 1, with *s* chosen so $s \le r$ and $(\tau -s)^2 + r^2= \tau ^2$, so $2 \tau s= r^2 + s^2 \le 2 r^2$, and hence $s \le r^2/\tau$. Thus $|C| \le 2 \theta _{d-1}r^{d+1}/\tau$, and Eq. 3.2 follows by letting $\tau \uparrow \tau (A)$. $\square$

In Lemma 3.6 below we give a lower bound on the volume within *A* of the difference between two balls, having their centres near the boundary of *A*.

#### Lemma 3.5

For any compact convex $F \subset \mathbb {R}^d$ containing a Euclidean ball of radius 1/4, any unit vector $e$ in $\mathbb {R}^d$, and any $a \in (0,2]$ we have $|(F + ae) \setminus F| \ge 8^{-d} \theta _{d-1} a$.

#### Proof

Without loss of generality, $F \supset B(o,1/4)$ and $e= e_d$. By Fubini’s theorem,

$$\begin{aligned} |(F + ae) \setminus F| \ge \int _{\pi (B(o,1/8))} \int {\textbf {1}}\{(u,t) \in F, (u,t+a)\notin F\} dt du. \end{aligned}$$

For any fixed $u\in \pi (B(o,1/8))$, by convexity the set of *t* such that the indicator is 1 is an interval of length at least $\min (a,1/4)$. Hence, the double integral is bounded from below by $\min (a,1/4) \theta _{d-1}8^{1-d}$. The result follows.$\square$

#### Lemma 3.6

If $r \in (0,\tau (A)/192)$ and $x, y \in A$ with $\Vert y-x \Vert \le 3r$ and $\,\textrm{dist}(x,\partial A) \le \,\textrm{dist}(y,\partial A)$, then

3.3 $$\begin{aligned} | A \cap B_r(y) \setminus B_r(x)| \ge 8^{-d} \theta _{d-1} r^{d-1} \Vert y-x\Vert . \end{aligned}$$

#### Proof

It suffices to consider the case with $x \in \partial A^{(r)} \cap A$. Let $x \in \partial A^{(r)} \cap A$. Without loss of generality (after a rotation and translation), we can assume that the closest point of $\partial A$ to *x* lies at the origin, and $x = \Vert x\Vert e_{d}$.

Fix $\tau \in (0,\tau (A))$. Since $z=o$ is the closest point in $\partial A$ to *x*, $\hat{n}_o = e_d$, so by the sphere condition $B_\tau (\tau e_d) \subset A$ and $B_\tau (-\tau e_d)^o \subset A^c$. For $u \in \mathbb {R}^{d-1}$ with $\Vert u\Vert < \tau$, define

$$ \phi (u):= \sup \{a \in [-\tau ,\tau ]: (u,a) \notin A\}. $$

Then $\phi (u) \le s(\Vert u\Vert )$ where for $0 \le v < \tau$ we define *s*(*v*) so $0 \le s(v) \le v$ and $(\tau - s(v))^2 + v^2 = \tau ^2$, and hence $s(v) \le v^2/\tau$ as in the proof of Lemma 3.4. Now suppose $0< r < \tau /4$. Set $K= 16/\tau$. Then

3.4 $$\begin{aligned} |\phi (u)| \le \tau ^{-1} \Vert u\Vert ^2 \le K r^2, ~~~ \forall u \in \mathbb {R}^{d-1} ~\textrm{with}~ \Vert u\Vert \le 4r. \end{aligned}$$

Let $y \in B_{3r}(x) \cap A \setminus \{x\}$ with $\,\textrm{dist}(y,\partial A) \ge \,\textrm{dist}(x,\partial A)$. We need to find a lower bound on $| A \cap B_r(y) \setminus B_r(x)|$.

First suppose $y \cdot e_d \ge x \cdot e_d$. Let $H:= \{z \in B_r(x): (z-x)\cdot e_d \ge r/4 \}$. We claim $H + (y-x) \subset A$. Indeed, for $z \in H + (y-x)$ we have $\Vert \pi (z)\Vert \le 4 r$, and hence $\phi (\pi (z)) \le Kr^2$ by Eq. 3.4. Therefore, provided $r<1/(4K)$, we have $z \cdot e_d \ge r/4 \ge Kr^2 \ge \phi (\pi (z))$, so $z \in A$, justifying the claim. Using the claim, and Lemma 3.5, we obtain that

3.5 $$\begin{aligned} | A \cap B_r(y) \setminus B_r(x) |&\ge |(H +(y-x)) \setminus H| \nonumber \\&\ge 8^{-d} \theta _{d-1} r^{d-1} \Vert y-x\Vert , ~\textrm{if}~ y \cdot e_d \ge x \cdot e_d. \end{aligned}$$

**Fig. 2:**
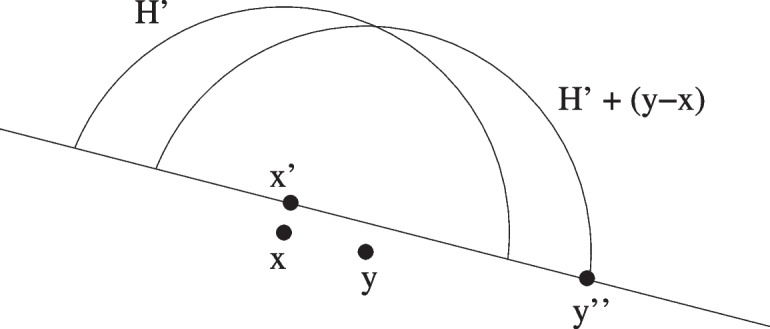
Illustration for proof of Lemma 3.6. The segment $H'$ is centred on *x*

Now suppose $y \cdot e_d < x \cdot e_d$. Note that $\pi (y) \ne \pi (x)$ since $y \ne x$ and $\,\textrm{dist}(y,\partial A) \ge \,\textrm{dist}(x,\partial A)$. Let $B'_r$ be the closed half-ball of radius *r* centred on *x*, having the property that $y':= x+ (r /\Vert y-x\Vert ) (y-x)$ has the lowest *d*-coordinate of all points in $B'_r$. Let $H'$ be the portion of $B_r(x)$ lying above the upward translate of the bounding hyperplane of $B'_r$ by a perpendicular distance of *r*/4 (see Fig. 2).

Since $\,\textrm{dist}(y,\partial A) \ge \,\textrm{dist}(x, \partial A) = x \cdot e_d$, using Eq. 3.4 we have

3.6 $$\begin{aligned} y \cdot e_d \ge \phi (\pi (y)) + x \cdot e_d \ge x \cdot e_d - (K/9)\Vert \pi (y)\Vert ^2. \end{aligned}$$

Let $x'$ be the point in the bounding hyperplane of $H'$ that lies closest to *x*. Then the line segment $[x,x']$ is almost vertical; the angle between this line segment and the vertical is the same as that between the line segment [*x*, *y*] and the horizontal. Therefore

3.7 $$\begin{aligned} \frac{(x' - x) \cdot e_d}{r/4} = \frac{\Vert \pi (y)\Vert }{\Vert y-x\Vert }. \end{aligned}$$

Using Eq. 3.6, provided $r < 1/K$ we have

$$ \Vert y-x\Vert \le \Vert \pi (y)\Vert (1 + (K/9) \Vert \pi (y)\Vert ) \le (9/8) \Vert \pi (y)\Vert , $$

so we obtain from Eq. 3.7 that

$$ (x' - x) \cdot e_d \ge (2/9) r. $$

Now letting $y''$ be the point in $H' + (y-x)$ with lowest *d*-coordinate, we have that $y'' = x' + a (y-x)$ with $a = 1 + (15/16)^{1/2}r/\Vert y-x\Vert$ (note $H'$ is not quite a half-ball). Hence

$$ (x'- y'') \cdot e_d \le \left( \frac{3r}{\Vert y-x\Vert } \right) (x-y) \cdot e_d \le K r^2, $$

where the last inequality came from Eq. 3.6. Hence, provided $r < 1 /(12K) = \tau /192$,

$$\begin{aligned} y''\cdot e_d = x'\cdot e_d - (x' - y'') \cdot e_d \ge x \cdot e_d + (2/9) r - K r^2 \ge r/8. \end{aligned}$$

On the other hand, for all $z \in H'+ (y-x)$ we have $\Vert \pi (z) \Vert \le 4r$, so that $\phi (\pi (z)) \le Kr^2$ by Eq. 3.4. Provided $r < 1/(8K)$ we therefore have $\phi (\pi (z)) \le r/8 \le z \cdot e_d$ and hence $z \in A$. Therefore $H' + (y-x) \subset A$. Also $H'$ contains a ball of radius *r*/4.

Therefore using Lemma 3.5, we have

$$ |A \cap B_r(y) \setminus B_r(x) | \ge |(H' + (y-x) ) \setminus H'| \ge 8^{-d} \theta _{d-1} r^{d-1}\Vert y-x\Vert ,~~\mathrm{if~} y\cdot e_d < x \cdot e_d. $$

Combined with Eq. 3.5 this yields Eq. 3.3.$\square$

### Integral Asymptotics

For $a \in [0,1]$, let $h(a):= |B_1(o) \cap ([0,a] \times \mathbb {R}^{d-1})|$ as at Eq. 3.1. The following lemma is very useful for estimating the integral of $\mathbb {P}[x \in V_{t,r,k}]$ over a region near the boundary of *A*, where $V_{t,r,k}$ was defined at Eq. 2.11.

#### Lemma 3.7

Let $\ell , j \in \mathbb {Z}_+:= \mathbb {N}\cup \{0\}$ and let $\alpha _0 >0$, $\varepsilon \in (0,1)$. Then as $s \rightarrow \infty$,

3.8 $$\begin{aligned} \theta _{d-1} \int _0^1 e^{-sh(a)} (\alpha _0 + h(a))^\ell da = \alpha _0^\ell s^{-1} + \ell \alpha _0^{\ell -1} s^{-2} + O(s^{\varepsilon -3}). \end{aligned}$$

Also

3.9 $$\begin{aligned} \theta _{d-1} \int _0^1 e^{-s h(a)} \left( \alpha _0 \!+\! h(a) \right) ^{j} \left( 1 + \frac{j}{s\left( \alpha _0 + h(a) \right) } \right) da = \alpha _0^j s^{-1} + 2 j\alpha _0^{j-1} s^{-2} + O(s^{\varepsilon -3}). \end{aligned}$$

#### Proof

Note first, for $0< x < 1$, that

$$\begin{aligned} h(x)&= \theta _{d-1} \int _0^x (1-y^2)^{(d-1)/2}dy = \theta _{d-1} \int _0^x \left( 1 + O (y^2) \right) dy \nonumber \\ &= \theta _{d-1}x + O(x^3) ~~~\textrm{as}~x \downarrow 0. \end{aligned}$$

Thus, setting $w=\theta _{d-1} sa$, we have $h(a) = w/s + O((w/s)^3)$, and $e^{-sh(a)} = e^{-w}(1+ O(w^3/s^2))$. Given $i \in \mathbb {Z}_+$, let $\delta = \varepsilon /(4+i)$. Then

$$\begin{aligned} \theta _{d-1} \int _0^{s^{\delta -1}} e^{-sh(a)} h(a)^i da&= \int _0^{\theta _{d-1}s^\delta } e^{-w} \Big (1 + O\big (\frac{w^3}{s^2}\big )\Big ) \Big (\frac{w^i}{s^{i+1}}\Big ) \Big (1 + O\big (\frac{w^2}{s^2}\big )\Big )^i dw \\&= s^{-i-1} \int _0^{\theta _{d-1}s^\delta } w^i e^{-w} dw + O\Big ( s^{-3-i} \int _0^{\theta _{d-1}s^{\delta }} w^{3+i} dw \Big ) \\&= s^{-i-1} \Big (i! - \int _{\theta _{d-1}s^\delta }^\infty w^i e^{-w} dw\Big ) + O(s^{-i-3} s^{\delta (4+i)}) \\&= i!s^{-i-1} + O(s^{\varepsilon -i-3}). \end{aligned}$$

Also $\int _{s^{\delta -1}}^1 e^{-sh(a)} h(a)^i da$ is $O(e^{-(\theta _{d-1}/2)s^\delta })$ since $(\theta _{d-1}/2) s^{\delta -1} \le h(a) \le \theta _d/2$ for *a* in this range. Therefore by binomial expansion, for $\ell \in \mathbb {Z}_+$ we have Eq. 3.8. Applying Eq. 3.8 with $\ell =j$ and (if $j >0$) also with $\ell = j-1$ gives us Eq. 3.9.$\square$

For integrating functions near the boundary of a smoothly-bounded set *A*, we have a useful change of variables which allows us to turn an integral over a region near the boundary into a double integral with one variable a boundary point and the other variable the distance to the boundary.

#### Proposition 3.8

(Reparameterization) There are positive finite constants $c = c(A), r_0 = r_0(A)$, such that for all $r \in (0,r_0)$, and all bounded measurable

$$\psi : A \rightarrow [0,\infty )$$,

3.10 $$\begin{aligned} \Big |\int _{\partial A^{(r)}} \psi (y) \, dy - \int _{0}^{r} \int _{\partial A} \psi (z + s \hat{n}_z) \, dz \, ds \Big | \le c r \int _{0}^{r} \int _{\partial A} \psi (z + s \hat{n}_z) \, dz \, ds, \end{aligned}$$

where the inner integral is a surface integral. If $\psi (y)$ depends only on $\,\textrm{dist}(y, \partial A)$, i.e. there exists $\Psi :[0,r_0) \rightarrow \mathbb {R}$ such that $\psi (z + s \hat{n}_z) = \Psi (s)$ for all $(z,s) \in \partial A \times (0,r_0]$, then

3.11 $$\begin{aligned} \Big | \int _{\partial A^{(r)}} \psi (y) \, dy - |\partial A| \int _0^{r} \Psi (s) \,ds \Big | \le c r |\partial A| \int _0^{r} \Psi (s) \,ds. \end{aligned}$$

#### Proof

By the assumptions on *A*, for each $x \in \partial A$ there is a constant $\delta (x) \in (0,\tau (A)/3)$, such that after a rotation $\mathcal {R}$ about *x*, within the ball $B(x,3 \delta (x))^o$, the set *A* coincides with the closed epigraph of a $C^{1,1}$ function $\phi :U\rightarrow \mathbb {R}$ with *U* an open ball of radius $3 \delta (x)$ in $\mathbb {R}^{d-1}$ centred on $\pi (x)$ (recalling that $\pi : \mathbb {R}^d \rightarrow \mathbb {R}^{d-1}$ denotes projection onto the first $d-1$ coordinates); that is,

$$ \mathcal {R}(A) \cap B(x,3 \delta (x)) = \{(u,s): u \in U, s \in [\phi (u),\infty ) \} \cap B(x,3 \delta (x)). $$

By a compactness argument we can cover $\partial A$ with a finite collection of balls $B(x_i, \delta (x_i))$, $1 \le i \le I$ with $x_1, \ldots , x_I \in \partial A$. For $r < \min _i \delta (x_i)$ we have $\partial A^{(r)} \subset \cup _{i=1}^I B(x_i,2 \delta (x_i))$. Since we can consider separately the integral of $\psi$ over $\partial A^{(r)} \cap B(x_1,2 \delta (x_1)$, over $\partial A^{(r)} \cap B(x_2,2 \delta (x_2)) \setminus B(x_1,2 \delta (x_1))$, over $\partial A^{(r)} \cap B(x_3,2 \delta (x_3)) \setminus [B(x_1,2 \delta (x_1)) \cup B(x_2,2 \delta (x_2))]$, and so on, it suffices to prove the result for the case where $\psi$ is supported by a single ball $B(x, 2 \delta (x))$ for some fixed $x \in \partial A$.

Without loss of generality we assume the rotation $\mathcal {R}$ is the identity map, so within the ball $B(x,3 \delta (x))^o$, *A* coincides with the closed epigraph of a $C^{1,1}$ function $\phi :U\rightarrow \mathbb {R}$ with *U* a $(d-1)$-dimensional open ball of radius $3 \delta (x)$ centred on $\pi (x)$.

**Fig. 3:**
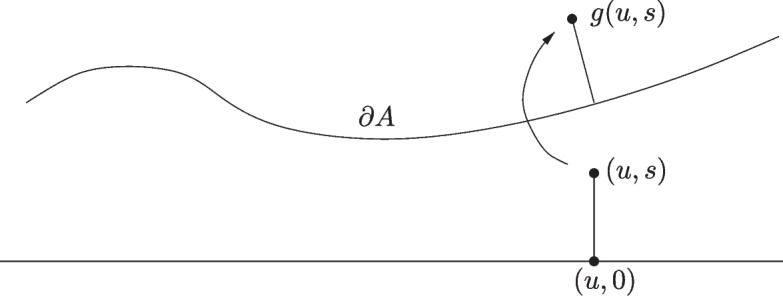
Illustration of the mapping *g* in the proof of Proposition 3.8

For $(u,s) \in U \times (0,\delta (x))$ let $g(u,s) := (u, \phi (u) ) + s \hat{n}_{(u,\phi (u))}$, as shown in Fig. 3, and observe that $\hat{n}_{(u,\phi (u))} = (1+ | \nabla \phi |^2)^{-1/2} (-\nabla \phi , 1).$ Since $\delta (x) < \tau (A)$, it follows from the sphere condition that $g: U \times (0,\delta (x)) \rightarrow A$ is injective. Since $\phi$ is $C^{1,1}$, $\nabla \phi (u)$ is Lipschitz continuous on $u \in U$, and therefore by Rademacher’s theorem (see e.g. Federer 1969), there exists a set $U' \subset U$ of full $(d-1)$-dimensional Lebesgue measure such that $\hat{n}_{u,\phi (u)}$ is differentiable for all $u \in U'$. Moreover by the Lipschitz continuity, and the definition of partial derivatives, all partial derivatives of $\hat{n}_{u,\phi (u)}$ are uniformly bounded on $U'$. Then for $0< r < \delta (x)$, by (Federer 1969, Theorems 3.2.5 and 2.10.43) (or if $\phi$ is $C^2$, (Billingsley 1979, Theorem 17.2) we have

3.12 $$\begin{aligned} \int _{\partial A^{(r)}} \psi (y)dy = \int _{U \times (0,r)} \psi (g(u,s)) \Big | \det \Big (\frac{\partial (g(u,s))}{\partial (u,s)} \Big ) \Big | d(u,s), \end{aligned}$$

where $J:= \frac{\partial g(u,s)}{\partial (u,s)}$ is the $d \times d$ Jacobian matrix of the mapping *g*, which is defined for all $(u,s) \in U' \times (0,r)$. Given $i,j \in \{1,\ldots , d-1\}$ the (*i*, *j*)th entry $J_{ij}$ of *J* is given by $\frac{\partial g_i}{\partial u_j} = \delta _{ij} + O(r)$, where the constant in the *O* term is independent of $u \in U'$ and $s \in (0,r)$. Also $J_{dj} = \frac{ \partial \phi }{\partial u_j} + O(r)$, while $J_{id} = \frac{ \partial g_i(u,s)}{\partial s}$ so the last column of *J* is given by the vector $\hat{n}_{(u,\phi (u))}$. Therefore

$$\begin{aligned} \Big | \det \Big (\frac{\partial (g(u,s))}{\partial (u,s)} \Big ) \Big |&= (1+ O(r))(1+ | \nabla \phi |^2)^{-1/2} \Big ( 1 + \big ( \frac{\partial \phi }{\partial u_1}\big )^2 + \cdots + \big (\frac{\partial \phi }{ \partial u_{d-1}}\big )^2 \Big ) \\&= (1+ O(r) )(1+ | \nabla \phi |^2)^{1/2}, \end{aligned}$$

where the *O* term is independent of $(u,s) \in U' \times (0,r)$. Therefore by Eq. 3.12,

$$\begin{aligned} \int _{A^{(r)}} \psi (y) dy&= (1+O(r)) \int _0^r \int _U \psi (g(u,s)) \sqrt{ 1 + |\nabla \phi (u)|^2} du ds \\&= (1+O(r)) \int _0^r \int _U \psi ((u,\phi (u))+ s\hat{n}_{(u,\phi (u))}) \sqrt{1 + |\nabla \phi (u)|^2} du ds \\&= (1+O(r)) \int _0^r \int _{\partial A} \psi (z + s \hat{n}_z) dz ds, \end{aligned}$$

which gives us Eq. 3.10. It is clear that Eq. 3.11 follows from Eq. 3.10. $\square$

## Probability Approximations

In this section we assume $k \in \mathbb {N}$ is fixed and $(r_t)_{t >0}$ is given and satisfies $tr_t^d = \Theta (\log t)$ as $t \rightarrow \infty$. With $V_{t,r,k}$ defined at Eq. 2.11, for $x, y \in A$ we define

4.1 $$\begin{aligned} p_t(x):= \mathbb {P}[x \in V_{t,r_t,k}]; ~~~~~ \pi _t(x,y):= \mathbb {P}[\{x,y\} \subset V_{t,r_t,k}]. \end{aligned}$$

Since *k* is fixed we are suppressing the dependence on *k* in this notation. For Borel $B \subset A$ with $|B|>0$, we define

4.2 $$\begin{aligned} \gamma _t(B) := (t/|B|) \mathbb {E}[|V_{t,r_t,k} \cap B|] = (t/|B|) \int _B p_t(x) dx, \end{aligned}$$

where the second identity in Eq. 4.2 comes from Fubini’s theorem.

In Lemma 4.1 below we approximate $\mathbb {P}[R'_{t,\tau t,k}(B) \le r_t]$ using Poisson approximation (by the Chen-Stein method) for the number of *Y*-points lying in the region $V_{t,r_t,k}$. Then in Lemma 4.2 we approximate $\mathbb {P}[R_{n,m,k}(B) \le r_n]$ by a suitable coupling of Poisson and binomial point processes.

### Lemma 4.1

(Poisson approximation) Suppose A1, A2 or A3 holds. Assume that $\gamma _t(B) = O(1)$ as $t \rightarrow \infty$. Let $\tau \in (0,\infty )$. Let $\varepsilon >0$. Then

$$ \sup _{\tau \in (\varepsilon ,1/\varepsilon )} |\mathbb {P}[R'_{t,\tau t,k}(B) \le r_t] - e^{-\tau \gamma _t(B)} | = O((\log t)^{1-d}). $$

### Proof

Let $$W_t:= \sum _{y \in \mathcal {Q}_{\tau t}} {\textbf {1}} \{\mathcal {P}_t(B_{r_t}(y)) < k\}.$$ Then $$\mathbb {P}[R'_{t,\tau t, k}(B) \le r_t] = \mathbb {P}[W_t =0]$$.

Let $d_{\textrm{TV}}$ denote total variation distance (see e.g. Penrose 2003). Then $|\mathbb {P}[ W_t =0 ]- e^{\tau \gamma _t(B)}| \le d_{\textrm{TV}}(W_t , Z_{\tau \gamma _t(B)})$. Hence, by a similar argument to (Penrose 2003, Theorem 6.7)

$$\begin{aligned} |\mathbb {P}[ W_t =0 ]- e^{\tau \gamma _t(B)}| \le 3 (I_1(t) + I_2(t)), \end{aligned}$$

where, with $p_t(x)$ and $\pi _t(x,y)$ defined at Eq. 4.1, we set

4.3 $$\begin{aligned} I_1(t)&:= \tau ^2 (t/|B|)^{2} \int _B \int _{B(x,3r_t) \cap B} p_t(x) p_t(y) dy dx; \\ I_2(t)&:= \tau ^2 (t/|B|)^{2} \int _B \int _{B(x,3r_t) \cap B} \pi _t(x,y) dy dx. \end{aligned}$$

Define the Borel measure $\nu$ on $\mathbb {R}^d$ by

4.4 $$\begin{aligned} \nu (\cdot ):= \lambda _d(\cdot \cap A)/|A|, \end{aligned}$$

where $\lambda _d$ denotes *d*-dimensional Lebesgue measure. Under any of A1, A2 or A3 (using Lemma 3.4 in the case of A1), we can and do choose $\delta >0$ such that for all $y \in B$ and all $r \in (0,1]$ we have $\nu (B_r(y) ) \ge 2 \delta r^d$. Hence, for all large enough *t* and all $y \in B$ we have

$$ p_t(y) = \sum _{j=0}^{k-1} ((t \nu (B_{r_t}(y)))^j/j!) e^{-t \nu (B_{r_t}(y))} \le \exp (-\delta tr_t^d). $$

Since $(t/|B|)\int _B p_t(x) dx = \gamma _t(B)$ which we assume is bounded, we have

4.5 $$\begin{aligned} I_1(t) \le \tau ^2 |B|^{-1} (t \theta _d (3r_t)^d) e^{-\delta tr_t^d} (t/|B|) \int _B p_t(x) dx = O(e^{-(\delta /2) t r_t^d}). \end{aligned}$$

Now consider $I_2(t)$. For $x,y \in A$ let us write $x \prec y$ if *x* is closer than *y* to $\partial A$ (in the Euclidean norm), or if *x* and *y* are the same distance from $\partial A$ but *x* precedes *y* lexicographically. Since $\pi _t(x,y) \textbf{1}\{\Vert y-x\Vert \le 3r_t\}$ is symmetric in *x* and *y*, we have

4.6 $$\begin{aligned} \int _B \int _{B(x,3r_t) \cap B } \pi _t(x,y) dy dx = 2 \int _B \int _{\{y \in B \cap B(x,3r_t): x \prec y\}} \pi _t(x,y) dy dx. \end{aligned}$$

By the independence properties of the Poisson process we have

$$ \pi _t(x,y) \le p_t(x) \sum _{m=0}^{k-1} q_{t,m}(y,x), $$

where we set $q_{t,m}(y,x) := \mathbb {P}[\mathcal {P}_t(B_{r_t}(y) \setminus B_{r_t}(x) ) = m].$

Suppose Assumption A1 or Assumption A3 applies. Set $\kappa _d:=8^{-d}\theta _{d-1}$. By Lemma 3.6, for all large enough *t* and all $x,y \in B$ with $x \prec y$ and $\Vert y-x\Vert \le 3r_t$, we have $\nu (B_{r_t}(y) \setminus B_{r_t}(x)) \ge \kappa _d f_0 r_t^{d-1} \Vert y-x\Vert$. Moreover by Fubini’s theorem $\nu (B_{r_t}(y) \setminus B_{r_t}(x)) \le \theta _{d-1}f_0 r_t^{d-1}\Vert y-x\Vert$. Hence for all $m \le k-1$,

$$ q_{t,m}(y,x) \le (t f_0 \theta _{d-1} r_t^{d-1}\Vert y-x\Vert )^m \exp (- \kappa _d f_0 t r_t^{d-1} \Vert y-x\Vert ). $$

Hence, setting $B_x:= \{y \in B: x \prec y\}$ we have that

$$\begin{aligned} t \int _{B_x \cap B(x,3r_t)} q_{t,m}(y,x)&\le f_0^m \theta _{d-1}^m t \int _{B(o,3r_t)} (t r_t^{d-1} \Vert y\Vert )^m \exp (-\kappa _d f_0 tr_t^{d-1} \Vert y\Vert ) dy \nonumber \\&= f_0^m \theta _{d-1}^m t (tr_t^{d-1})^{-d} \int _{B(o,3tr_t^d)} \Vert z\Vert ^m \exp (- \kappa _d f_0 \Vert z\Vert ) dz \\&\le c(tr_t^d)^{1-d}, \end{aligned}$$

for some constant *c* depending only on $$d, f_0 $$ and *k*. Therefore

$$\begin{aligned} t^2 \int _B \int _{B(x,3r_t) \cap B} \pi _t(x,y) dy dx \le 2 \left( t \int _B p_t(x) dx \right) ck (tr_t^d)^{1-d}. \end{aligned}$$

Since the expression in brackets on the right is *O*(1) by assumption, we thus have $I_2(t) =O((tr_t^d)^{1-d}) = O((\log t)^{1-d})$.

Now suppose instead that Assumption A2 applies. First we examine the situation where *x* is not too close to the corners of *A*. Suppose that $\,\textrm{dist}(x,\Phi _0(A))>Kr_t$, where $\Phi _0(A)$ denotes the set of corners of *A* and the constant *K* will be made explicit later. We can assume that the corner of *A* closest to *x* is formed by edges $e,e'$ meeting at the origin with angle $\alpha \in (0,2\pi ) \setminus \{\pi \}$. We claim that, provided $K>4+8/|\sin \alpha |$, the disk $B(x,4r_t )$ intersects at most one of the two edges. Indeed, if it intersects both edges, then taking $w\in B(x,4r_t) \cap e, w'\in B(x,4r_t) \cap e'$ we have $\Vert w-w'\Vert \le 8r_t$; hence $\,\textrm{dist}(w,e')\le 8r_t$. Then, $\Vert w\Vert = \,\textrm{dist}(w,e')/|\sin \alpha |\le 8r_t /|\sin \alpha |$. However, $\Vert w\Vert \ge (K-4)r_t$ by the triangle inequality, so we arrive at a contradiction. Also, for *t* sufficiently large, non-overlapping edges of *A* are distant more than $8r_t$ from each other. We have thus shown that if we take $K = 5 +(8/\min _i |\sin \alpha _i|)$, where $\{\alpha _i\}$ are the angles of the corners of *A*, then for large *t*, no ball of radius $4r_t$ distant at least $Kr_t$ from the corners of *A* can intersect two or more edges of *A* at the same time.

We have $B(x,r_t)\cup B(y,r_t)\subset B(x,4r_t)$. Hence, the argument leading to Lemma 3.6, shows that $\nu (B(y,r_t) \setminus B(x,r_t)) \ge \theta _{d-1} 8^{-d} f_0 \Vert x-y\Vert r_t$. Using this, we can estimate the contribution to the double integral on the right side of Eq. 4.7 in the same way as we did under assumption A1.

Suppose instead that *x* is close to a corner of *A* and $\Vert x-y\Vert \le 3r_t$. The contribution to the double integral on the right side of Eq. 4.7 from such pairs (*x*, *y*) is at most $c'' t^2 r_t^4 \exp (-\delta _1 tr_t^2)$ where $c''$ depends only on *K* and $\delta _1>0$ depends only on *A*. Therefore this contribution tends to zero, and the proof is now complete.$\square$

### Lemma 4.2

(De-Poissonization) Suppose A1, A2 or A3 holds. Let *m*(*n*) be such that $m(n) = \Theta (n)$ as $n \rightarrow \infty$. Assume $\gamma _n(B) = O(1)$ as $n \rightarrow \infty$. Then

$$ |\mathbb {P}[R_{n,m(n),k}(B) \le r_n] - e^{-(m(n)/n) \gamma _n(B)} | = O((\log n)^{1-d}). $$

### Proof

Write *m* for *m*(*n*). Set $n^+:= n+ n^{3/4}$, $n^-:= n- n^{3/4}$ and $m^+:= m+ m^{3/4}, m^-:= m-m^{3/4}$. Set

$$ W := \sum _{y \in \mathcal {Y}_m} {\textbf {1}} \{\mathcal {X}_n(B_{r_n}(y))< k\}; ~~~ W' := \sum _{y \in \mathcal {Q}_{m^-,B}} {\textbf {1}} \{\mathcal {P}_{n^+}(B_{r_n}(y)) <k\}. $$

Set $\gamma '_n: = (n^-/|B|) \mathbb {E}[|V_{{n^+},r_n,k} \cap B|]$, where $V_{n,r,k}$ was defined at Eq. 2.11. Then, with the measure $\nu$ defined at Eq. 4.5,

4.7 $$\begin{aligned} \gamma '_n&= \frac{n^-}{|B|} \int _B \left( e^{-n \nu (B(x,r_n)) - n^{3/4} \nu (B(x,r_n))} \sum _{j=0}^{k-1} (n (1+n^{-1/4}))^j\nu (B(x,r_n))^j/j! \right) dx \nonumber \\&= \gamma _n(B)(1+ O((\log n)^{1/d}n^{-1/4})). \end{aligned}$$

By Lemma 4.1, $|\mathbb {P}[R'_{n^+,m^-,k} (B) \le r_n]-e^{-(m^-/n^+)\gamma '_n}| = O((\log n)^{1-d})$, and hence by Eq. 4.8, $|\mathbb {P}[ R'_{n^+,m^-,k}(B) \le r_n] -e^{-(m^-/n^+)\gamma _n(B)}| = O((\log n)^{1-d})$. Note also that $|(m/n)-(m^-/n^+)|= O(n^{-1/4})$ so that $|e^{-(m/n)\gamma _n(B)}-e^{-(m^-/n^+)\gamma _n(B)}| = O(n^{-1/4})$. Also

$$\begin{aligned} | \mathbb {P}[R_{n,m(n),k}(B) \le r_n] - \mathbb {P}[R'_{n^+,m^-,k}(B) \le r_n] | \le \mathbb {P}[W \ne W']. \end{aligned}$$

We have the event inclusion $\{W \ne W'\} \subset E_1 \cup E_2 \cup E_3$, where, recalling the definition of $(Z_t)_{t \ge 0}$ in Section 1, we define the events

$$\begin{aligned} E_1&:= \{Z_{m^-} \le m \le Z_{m^+}\}^c \cup \{Z'_{n^-} \le n \le Z'_{n^+}\}^c; \\ E_2&:= \{ \exists y \in \mathcal {Q}_{m^-}: \mathcal {P}_{n^-}(B(y,r_n))< k, (\mathcal {P}_{n^+} \setminus \mathcal {P}_{n^-}) (B(y,r_n)) \ne 0\}; \\ E_3&:= \{ \exists y \in \mathcal {Q}_{m^+} \setminus \mathcal {Q}_{m^-}: \mathcal {P}_{n^-}(B(y,r_n)) < k \}. \end{aligned}$$

By Chebyshev’s inequality $\mathbb {P}[E_1] = O(n^{-1/2})$. Also by a similar calculation to Eq. 4.8, $(n/|B|)\mathbb {E}[|V_{n^-,r_n,k}\cap B|] = \gamma _n(B)(1+ O((\log n)n^{-1/4}))$, and

$$\begin{aligned} \mathbb {P}[E_2] \le \mathbb {E}[|V_{n^-,r_n,k} \cap B|/|B|] m^- (2 n^{3/4}) f_0 \theta _d r_n^d = O(n^{-1/4} \log n). \end{aligned}$$

Similarly $\mathbb {P}[E_3] = 2m^{3/4} \mathbb {E}[|V_{n^-,r_n,k} \cap B|/|B|] = O(n^{-1/4})$. Combining these estimates gives the result.$\square$

## Proof of Theorems

### Proof of Theorem 2.1

Recall the definition of $\gamma _{t}(B)$ at Eq. 4.2. For each theorem, we need to find $(r_t)_{t \ge 0}$ such that $\gamma _t(B)$ converges as $t \rightarrow \infty$; we can then apply Lemmas 4.1 and 4.2. We are ready to do this for Theorem 2.1 without further ado. Recall that $f_0:=1/|A|$.

#### Proof of Theorem 2.1

Fix $k \in \mathbb {N}$, $\beta \in \mathbb {R}$. For all $t>0$ define $r_t \in [0,\infty )$ by

$$\begin{aligned} t f_0 \theta _d r_t^d = \max (\log t + (k-1) \log \log t + \beta ,0). \end{aligned}$$

Set $j=k-1$. Since we assume here that $\overline{B} \subset A^o$, for *t* large we have for all $x \in B$ that $B(x,r_t) \subset A$, and hence by (Last and Penrose 2018, Theorem 1.3), $\mathcal {P}_t(B(x,r_t))$ is Poisson with parameter $t |B(x,r_t)|/|A| = t f_0 \theta _d r_t^d$. By Eq. 2.11, $\mathbb {P}[x \in V_{t,r_t,k}] = \mathbb {P}[\mathcal {P}_t(B(x,r_t)) < k]$, so as $t \rightarrow \infty$ we have uniformly over $x \in B$ that

4.8 $$\begin{aligned} \mathbb {P}[x \in V_{t,r_t,k}]&= e^{-tf_0\theta _dr_t^d}( (t f_0 \theta _dr_t^d)^j/j!) (1+ j (tf_0 \theta _d r_t^d)^{-1} + O((\log t)^{-2})) \\&= (1/j!) t^{-1} (\log t)^{-j} e^{-\beta } (\log t + j \log \log t + \beta )^j \nonumber \\&~~~~~~~~~~~~~~\times (1+ j (\log t + j \log \log t + \beta )^{-1} +O((\log t)^{-2})) \nonumber \\&= \frac{e^{-\beta }}{j!t} \Big ( 1+ \frac{j \log \log t + \beta }{\log t} \Big )^j \Big (1 + j (\log t)^{-1} \Big ( 1+ \frac{j \log \log t + \beta }{ \log t} \Big )^{-1} \nonumber \\&~~~~~~~~~~~~~~~~~~~~~~~~~~~~~~~ ~~~~~~~~~~~~~~~~~~~~~~~~~~~~~~ + O \big ((\log t)^{-2} \big ) \Big ), \nonumber \end{aligned}$$

where the $O(\cdot )$ term is zero for $k=1$ or $k=2$. Using Eq. 4.2, we obtain by standard power series expansion that as $t \rightarrow \infty$ we have

5.1 $$\begin{aligned} \gamma _t(B)&= \frac{e^{-\beta }}{j!} \Big ( 1 + \frac{j^2 \log \log t}{\log t} + \frac{j(1+\beta )}{\log t} + O\big ( \big ( \frac{\log \log t}{\log t} \big )^2 \big ) \Big ). \end{aligned}$$

Hence by Lemma 4.1, given $\tau \in (0,\infty )$ as $t \rightarrow \infty$ we have

5.2 $$\begin{aligned} \mathbb {P}[R'_{t,\tau t,k}(B) \le r_t ] = \exp \Big (-\frac{\tau e^{-\beta }}{j!} \Big ( 1 + \frac{j^2 \log \log t}{\log t} + \frac{j(1+\beta )}{\log t} + O\big ( \big ( \frac{\log \log t}{\log t} \big )^2 \big ) \Big ) \Big ) \nonumber \\ ~~~~~~~~~~~~~~~~~~~~~~~ ~~~~~~~~~~~~~~~~~~~~~~~ ~~~~~~~~~~~~~~~~~~~~~~~ + O((\log t)^{1-d}) \nonumber \\ = {\left\{ \begin{array}{ll} e^{- \tau e^{-\beta }/j!} \left( \exp \big ( - \frac{\tau e^{-\beta } j^2 \log \log t}{j! \log t} \big ) + O\big ( (\log t)^{-1} \big ) \right) ~~~~~~~~~~~~~~~~~ ~~~ \textrm{if}~d=2 \\ e^{- \tau e^{-\beta }/j!} \left( \exp \big (- \frac{\tau e^{-\beta } j^2 \log \log t}{j! \log t} - \frac{\tau e^{-\beta } j(1+\beta )}{j! \log t} \big ) + O\big ( \big (\frac{\log \log t}{\log t} \big )^2 \big ) \right) ~~~\textrm{if}~d \ge 3, \end{array}\right. } \end{aligned}$$

yielding Eq. 2.3. Similarly, given also $m: \mathbb {N}\rightarrow \mathbb {N}$ satisfying $\tau _n:= m(n)/n \rightarrow \tau$ as $n \rightarrow \infty$, by Lemma 4.2 and Eq. 5.2 we have as $n \rightarrow \infty$ that

5.3 $$\begin{aligned}&\mathbb {P}[R_{n,m(n),k}(B) \le r_n ] \nonumber \\&= {\left\{ \begin{array}{ll} e^{- \tau _n e^{-\beta }/j!} \exp \Big ( - \frac{\tau _n e^{-\beta } j^2 \log \log n}{j! \log n} \Big ) + O\big ( (\log n)^{-1} \big ) ~~~~~~~~~~~~~~~~~~ ~~~ \textrm{if}~d=2 \\ e^{- \tau _n e^{-\beta }/j!} \exp \Big ( - \frac{\tau _n e^{-\beta } j^2 \log \log n}{j! \log n} - \frac{\tau _n e^{-\beta } j(1+\beta )}{j! \log n} \Big ) + O\big ( \big (\frac{\log \log n}{\log n} \big )^2 \big ) ~~~\textrm{if}~d \ge 3, \end{array}\right. } \end{aligned}$$

and Eq. 2.2 follows.$\square$

Under assumption A1 or A2 (with $B=A$), it takes more work than in the preceding proof to determine $r_t$ such that $\gamma _t(A)$ tends to a finite limit. The right choice turns out to be as follows. Let $\beta \in \mathbb {R}$ and let $r_t=r_t(\beta )\ge 0$ be given by

5.4 $$\begin{aligned} f_0 t\theta _d r_t^d = \max \Big ( \big (2- 2/d \big ) \log t + \big ( 2k -4 + 2/d \big ) J(d,k) \log \log t + \beta , 0 \Big ), \end{aligned}$$

where $f_0:= |A|^{-1}$ and $J(d,k) := {\textbf {1}}_{\{d\ge 3 \text { or } k\ge 2\}}$. We show in the next subsection that this choice of $r_t$ works.

### Convergence of $t \mathbb {E}[|V_{t,r_t,k}|]$

Recall $p_{t}(x)$ at Eq. 4.1. By Fubini’s theorem, as at Eq. 4.2, we have

5.5 $$\begin{aligned} \mathbb {E}[|V_{t,r_t,k}|]= \int _A p_{t}(x) dx. \end{aligned}$$

Recalling the notation $$\partial A^{(r)}$$ and $$A^{(-r)}$$ from the start of Section 3, we refer to the region $$ A^{(-r_t)}$$ as the *bulk*, and the region $$\textsf{Mo}_{t}:= \partial A^{(r_t)}$$ as the *moat*.

#### Proposition 5.1

(Convergence of the expectation when $$d \ge 3$$) Suppose Assumption A1 applies with $$d \ge 3$$. Fix $$\beta \in \mathbb {R}, \varepsilon >0$$ and let $$r_t := r_t(\beta ), V_{t,r,k} $$ and $$ c_{d,k}$$ be as given in Eqs. 5.5, 2.11 and 2.6. Then as $$t \rightarrow \infty $$,

5.6 $$\begin{aligned} t \mathbb {E}[|V_{t,r_t,k}|]&= e^{-\beta /2} c_{d,k} f_0^{-1/d} |\partial A| \Big (1 + \frac{ (k-2 + 1/d)^2 \log \log t}{(1-1/d)\log t} \nonumber \\&~~~~~~~~~~~~~ + \frac{ (k-2 + 1/d) \beta + 4k -4}{(2-2/d) \log t} \Big ) + O\big ( (\log t)^{\varepsilon -2} \big ). \end{aligned}$$

To prove this, we shall investigate separately the contributions to the integral in the right hand side of Eq. 5.6 from the the different regions $A^{(-r_t)}$ and $\textsf{Mo}_t$ of the set *A* (it turns out that when $d \ge 3$, the main contribution always comes from the moat regardless of *k*.) To avoid repeating ourselves later on, we shall deal with $\textsf{Mo}_t$ in a manner that covers the case $d=2$ as well.

#### Lemma 5.2

(Contribution of the moat) Suppose Assumption A1 applies with $d \ge 2$. Fix $\beta \in \mathbb {R}, \varepsilon >0$ and let $r_t:= r_t(\beta )$ be given by Eq. 5.5. Set $J'(d,k):=1 - J(d,k)$. Then

5.7 $$\begin{aligned} t \int _{\textsf{Mo}_{t}} p_{t}(x) dx =&c_{d,k} f_0^{-1/d}e^{-\beta /2} |\partial A| (\log t)^{-\frac{1}{2} J'(d,k)} \Big (1+ \frac{(k-2+1/d)^2 J(d,k) \log \log t}{(1-1/d) \log t} \nonumber \\&~~~~~ + \frac{ (k-2+ 1/d) \beta + \ 4k -4}{(2-2/d) \log t} + O\big ( (\log t)^{\varepsilon -2} \big ) \Big ). \end{aligned}$$

#### Proof

Given $t >0$, $x \in A$ set $\mu _t(x):= t f_0 |B_{r_t}(x) \cap A|$ and let $a(x):= \,\textrm{dist}(x,\partial A)$. Then by Eq. 4.1, $p_t(x) = \mathbb {P}[Z_{\mu _t} <k]$, where $Z_u \sim$ Poisson(*u*). Also for *t* large we have $\mu _t(x) \ge 1$ for all $x \in A$. Hence, similarly to Eq. 5.1 we have uniformly over $x \in A$ that

5.8 $$\begin{aligned} p_{t}(x)&= ((k-1)!)^{-1} e^{- \mu _{t}(x) } \mu _{t}(x)^{k-1} (1+ ((k-1)/ \mu _t(x)) + O((\mu _t(x))^{-2})). \end{aligned}$$

For $a \in (0,1]$ set $\Lambda _{t,a}:= tf_0 r_t^d(\frac{1}{2} \theta _d + h(a))$, with $h(\cdot )$ defined at Eq. 3.1. By Lemma 3.4 and Eq. 5.5, we have that

$$ \sup _{x \in \textsf{Mo}_t} |\mu _t(x) - \Lambda _{t,a(x)/r_t} | = O(t r_t^{d+1}) = O \Big ( (\log t)^{(d+1)/d} t^{-1/d} \Big ) $$

where we have used also the fact that $tr_t^d = \Theta (\log t)$. Also $\Lambda _{t,a}=\Theta (\log t)$ uniformly over $a\in [0,1]$. Hence, for each $x\in \textsf{Mo}_{t}$, by Eq. 5.9 we have

$$\begin{aligned} p_t(x) = ((k-1)!)^{-1} e^{- \Lambda _{t,a(x)/r_t} } \Lambda _{t,a(x)/r_t}^{k-1} (1+( (k-1)/\Lambda _{t,a(x)/r_t}) + O((\log t)^{-2})), \end{aligned}$$

where the constant in the *O* term is independent of *x*. Then by Proposition 3.8,

$$\begin{aligned} t \int _{\textsf{Mo}_{t}} p_{t}(x)dx = \left( \frac{t |\partial A|(1+ O(r_t))}{(k-1)!} \right) r_t \int _0^1 e^{-\Lambda _{t,a}} \Lambda _{t,a}^{k-1} \Big (1+ \frac{k-1}{\Lambda _{t,a}} + O\big ((\log t)^{-2}\big ) \Big )da. \end{aligned}$$

By Eq. 5.5, for *t* large $e^{-tf_0\theta _d r_t^d/2}= t^{-(1-1/d)} (\log t)^{(2-k-1/d)J(d,k)} e^{-\beta /2}$, and setting $s = tf_0r_t^d$ we have $\Lambda _{t,a}= s(\frac{1}{2} \theta _d + h(a))$, so that

$$\begin{aligned} t \int _{\textsf{Mo}_{t}} p_{t}(x)dx&= \Big ( \frac{ t |\partial A| r_t}{(k-1)!} \Big ) t^{-(1-1/d)} (\log t)^{(2-k-1/d)J(d,k)} e^{-\beta /2} s^{k-1} \\&\times \int _0^1 e^{-sh(a)} \left( \frac{\theta _d}{2} + h(a) \right) ^{k-1} \left( 1+ \frac{k-1}{s(\frac{\theta _d}{2}+ h(a))} + O((\log t)^{-2}) \right) da. \end{aligned}$$

Hence by Lemma 3.7 with $\alpha _0= \theta _d/2$ and $j=k-1$, given $\varepsilon >0$ we have

$$\begin{aligned}&t \int _{\textsf{Mo}_{t}} p_{t}(x)dx = \Big ( \frac{ t |\partial A| r_t}{(k-1)!} \Big ) t^{-(1-1/d)} (\log t)^{(2-k-1/d)J(d,k)} e^{-\beta /2} s^{k-1} \\&~~~~~~~~~~~~~~~~~~~~~~~~~~~~~~~ \times \frac{(\theta _d/2)^{k-1}}{\theta _{d-1}s} \left( 1 + 4(k-1)\theta _d^{-1} s^{-1} + O(s^{\varepsilon -2}) \right) \\&\!=\! \frac{ f_0^{-1/d} \theta _d^{k-1} |\partial A| e^{-\beta /2} }{(k-1)!2^{k-1} \theta _{d-1}} \left( \frac{s}{\log t} \right) ^{k-2+1/d} (\log t)^{-\frac{1}{2} J'(d,k)} ( 1 \!+\! 4(k-1)\theta _d^{-1} s^{-1} + O(s^{\varepsilon -2})). \end{aligned}$$

By Eq. 5.5, for *t* large we have

$$ s= \frac{(2-2/d) \log t}{\theta _d} \left( 1 + \frac{(k -2 + 1/d) J(d,k) \log \log t + \beta /2 }{(1-1/d)\log t} \right) . $$

Therefore

5.9 $$\begin{aligned} t \int _{\textsf{Mo}_{t}} p_{t}(x) dx = \frac{ f_0^{-1/d} \theta _d^{k-1} |\partial A| e^{-\beta /2} }{(k-1)!2^{k-1} \theta _{d-1}} \left( \frac{2-2/d}{\theta _d} \right) ^{k-2+1/d} (\log t)^{-\frac{1}{2} J'(d,k)} ~~~~~~~~~~~~~~~ \nonumber \\ \times \left( 1 + \frac{(k-2+1/d) ((k-2 + 1/d)J(d,k) \log \log t + \beta /2)}{ (1- 1/d)\log t} +O\big (\big ( \frac{\log \log t}{\log t}\big )^2\big ) \right) \nonumber \\ \times \left( 1 + \frac{4k-4 }{(2-2/d) \log t } + O \big ( (\log t)^{\varepsilon -2} \big )\right) , \nonumber \end{aligned}$$

and therefore by the definition Eq. 2.6 of $c_{d,k}$, Eq. 5.8 holds.$\square$

#### Proof of Proposition 5.1

To deal with the bulk, we use Eq. 5.9, noting that for $x \in A^{(-r_t(\beta ))}$ we have $\mu _t(x) = t f_0 \theta _d r_t^d$. Hence for such *x* we have

$$ p_t(x) = O((t r_t^d)^{k-1} e^{-t f_0 \theta _d r_t^d}) = O( ( \log t)^{k-1} t^{-(2-2/d)} (\log t)^{4- 2k-2/d} ), $$

where the constant in the *O* term does not depend on *x*, so that

5.10 $$\begin{aligned} t \int _{A^{(-r_t)}} p_t(x)dx = O( (\log t)^{2} t^{-1+ 2/d }). \end{aligned}$$

Using Eqs. 5.8, 5.10 and 5.6, we obtain Eq. 5.7 for $d \ge 3$ as required.$\square$

#### Proposition 5.3

(Convergence of the expectation when $d=2$) Suppose $d=2$ and A1 or A2 applies. Fix $\beta \in \mathbb {R}$, and let $r_t, V_{t,r,k}$ be as given in Eqs. 5.5 and 2.11. Then as $t \rightarrow \infty$,

5.11 $$\begin{aligned} t\mathbb {E}[|V_{t,r_t,k}|] = {\left\{ \begin{array}{ll} |A| e^{-\beta } + |\partial A|e^{-\beta /2} \frac{\sqrt{\pi /f_0}}{2} (\frac{1}{\sqrt{\log t}} + O((\log t)^{-3/2}) ) & \text{ if } k= 1 \\ |A| e^{-\beta } + |\partial A|e^{-\beta /2} \frac{\sqrt{\pi /f_0}}{4} \Big ( 1 + \frac{\log \log t}{2 \log t} \Big ) + \frac{|A|e^{-\beta }\log \log t}{\log t} \\ ~~~~~~~~~~~~~~~~~~~~~~~~~~~~ ~~~~~~~~~~~+ O((\log t)^{-1}) ) & \text{ if } k=2 \\ |\partial A|e^{-\beta /2} \frac{\sqrt{\pi /f_0}}{(k-1)!2^k} \left( 1+\frac{(2k-3)^2\log \log t }{2 \log t} \right) + O\big (\frac{1}{\log t}\big ) & \text{ if } k\ge 3. \end{array}\right. } \end{aligned}$$

#### Proof

**Case 1:** *A* **has a** $C^{1,1}$ **boundary and** $\overline{A^o}=A$. We first estimate the contribution to Eq. 5.6 from the bulk. By Eq. 5.9, for $x\in A^{(-r_t)}$ we have

$$ p_{t}(x) = \frac{1}{(k-1)!} e^{ - tf_0\pi r_t^2} (tf_0\pi r_t^2)^{k-1} \Big (1+ \frac{k-1}{tf_0\pi r_t^2} + O(\frac{1}{(tr_t^2)^2}) \Big ), $$

where the O term is 0 when $k=1$ or $k=2$. Also by Eq. 5.5, for *t* large we have $e^{-tf_0 \pi r_t^2}= e^{-\beta }t^{-1} (\log t)^{(3-2k){\textbf {1}}_{\{k \ge 2\}}}$. Hence for $k \ge 2$ and $x \in A^{(-r_t)}$ we have

$$\begin{aligned} p_{t}(x) = (t^{-1} e^{-\beta }/(k-1)!) (\log t)^{3-2k} (\log t + (2k-3)\log \log t + \beta )^{k-1} \\ \times (1+ (k-1)(\log t)^{-1}(1+ O((\log \log t)/\log t))), \end{aligned}$$

while if $k=1$ and $x \in A^{(-r_t)}$ then $p_t(x) = e^{-\beta }t^{-1}$. Hence, since $|A^{(-r_t)}| = |A_t| + O(r_t)$,

5.12 $$\begin{aligned} \int _{A^{(-r_t)}} p_{t}(x) tdx ={\left\{ \begin{array}{ll} e^{-\beta } |A| (1+ O(r_t)) & \text{ if } k=1, \\ e^{-\beta } |A| \big (1+\frac{\log \log t}{\log t} + \frac{\beta + 1}{\log t} + O( \frac{\log \log t}{(\log t)^2}) \big ) & \text{ if } k=2, \\ \frac{e^{-\beta }}{(k-1)!(\log t)^{k-2}} + O(\frac{\log \log t}{ (\log t)^{k-1}})& \text{ if } k\ge 3. \end{array}\right. } \end{aligned}$$

For the contribution from the moat, we use Lemma 5.2. Note that $c_{2,k} = \frac{\pi ^{1/2}}{(k-1)!2^k}$. By Eq. 5.8,

5.13 $$\begin{aligned} t \int _{\textsf{Mo}_t}p_{t}(x) dx = {\left\{ \begin{array}{ll} \frac{1}{2} |\partial A| e^{-\beta /2} \sqrt{\pi /f_0} ((\log t)^{-1/2} + O((\log t)^{-3/2})) & \textrm{if}~ k=1, \\ |\partial A| e^{-\beta /2} \frac{\sqrt{\pi /f_0}}{(k-1)!2^k} \Big (1 + \frac{(2k-3)^2\log \log t }{2 \log t } \Big ) + O\big (\frac{1}{\log t} \big ) & \textrm{if}~ k\ge 2. \end{array}\right. } \end{aligned}$$

Combining this with Eqs. 5.12, and using 5.6, yields Eq. 5.11 in Case 1.

**Case 2:** *A* **is polygonal.** In this case, the contribution of the bulk $A^{(-r_t)}$ to the integral on the right hand side of Eq. 5.6 can be dealt with just as in Case 1; that is, Eq. 5.12 remains valid in this case.

**Fig. 4:**
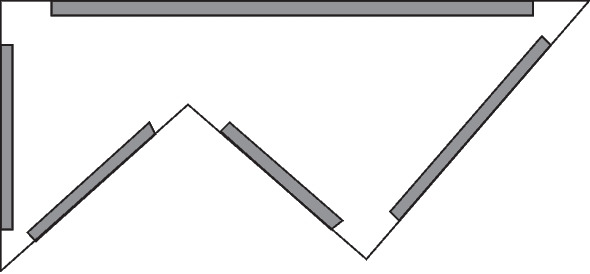
Illustration showing the rectangles $\textsf{Rec}_{t,i}$ (shaded) in the proof of Proposition 5.3, Case 2

Let $|\Phi _0(A)|$ denote the number of corners of *A* and enumerate the edges and corners of *A* in some arbitrary order. For $1 \le i \le |\Phi _0(A)|$, let $\textsf{Rec}_{t,i}$ denote a rectangular region in *A* having as its base part of the *i*th edge of *A*. We take each rectangle to have width $r_t$ and each end of each rectangle to be distant $K r_t$ from the corner of *A* at the corresponding end of the corresponding edge of *A*, with *K* chosen large enough so that $K> 3/|\sin (\alpha /2)|$ for each angle $\alpha$ of the polygon *A*, as shown in Fig. 4. This choice of *K* ensures that the rectangular regions are pairwise disjoint.

We define the corner region $\textsf{Cor}_{t} := \textsf{Mo}_t \setminus \cup _{i=1}^{|\Phi _0(A|} \textsf{Rec}_{t,i}$. Let $\textsf{Cor}_{t,i}$ denote the intersection of *A* with the disk of radius $(K+1)r_t$ centred on the *i*th corner of *A*. Then $\textsf{Cor}_t \subset \cup _{i=1}^{|\Phi _0(A)|}\textsf{Cor}_{t,i}$.

Then there exists $\kappa >0$ (depending on the sharpest angle of *A*), such that for all large *t* and any $i \le |\Phi _0(A)|$, $x\in \textsf{Cor}_{t,i}$, we have $p_t(x)\le e^{- tf_0 \pi r_t^2 \kappa }$ and hence by Eq. 5.5, $p_t(x) \le t^{-\kappa }$ (note that the coefficient of $\log \log t$ in Eq. 5.5 is nonnegative in this case). Each $\textsf{Cor}_{t,i}$ has area at most $\pi ((K+1)r_t)^2$, and hence

5.14 $$\begin{aligned} t f_0 \int _\mathsf{{Cor}_{t,i}} p_t(x)dx = O( t r_t^2 t^{-\kappa } ) = O( (\log t) t^{-\kappa }), \end{aligned}$$

leading to a total contribution from the corners to Eq. 5.6 of $O((\log t)t^{-\kappa })$. Since there are a fixed number of corner regions, so the total contribution of these regions to the integral on the right hand side of Eq. 5.6 is $O((\log t)t^{-\kappa })$.

By the proof of Lemma 5.2 we see that the total contribution of the rectangles $\textsf{Rec}_{t,i}, 1 \le i \le |\Phi _0(A)|$, to the right hand side of Eq. 5.6 is the same as Eq. 5.13. There is an extra multiplicative error term of $O(r_t)$ due to the total length of the rectangles being less than the perimeter of *A*, but this error term is dominated by the error terms already included in Eq. 5.13.

Putting together these estimates yields Eq. 5.11 in Case 2.$\square$

### Proof of theorems 2.2 and 2.3

#### Proof of Theorem 2.2

Recall $f_0:= 1/|A|$. Suppose $(r_t)_{t>0}$ satisfies the case $d=2,k=1$ of Eq. 5.5, so that $t \pi f_0 r_t^2 - \log t = \beta$ for all large enough *t*. Then by Eq. 4.2, Proposition 5.3 and Eq. 2.1,

$$\begin{aligned} \gamma _t(A):= t f_0 \mathbb {E}[|V_{t,r_t}|] = e^{-\beta } + (\sigma _A e^{-\beta /2} \pi ^{1/2}/2) (\log t)^{-1/2} + O((\log t)^{-3/2}). \end{aligned}$$

Hence by Lemma 4.1,

$$\begin{aligned} \mathbb {P}[R'_{t,\tau t} \le r_t] = \exp \big ( - \tau e^{-\beta } - \tau (\sigma _Ae^{-\beta /2} \pi ^{1/2}/2) (\log t)^{-1/2} \big ) + O((\log t)^{-1}), \end{aligned}$$

and hence Eq. 2.5. Also by Lemma 4.2, setting $\tau _n = m(n)/n$ we have

$$\begin{aligned} \mathbb {P}[R_{n,m } \le r_n] = \exp \big ( - \tau _n e^{-\beta } - \tau _n (\sigma _Ae^{-\beta /2} \pi ^{1/2}/2) (\log n)^{-1/2} \big ) + O((\log n)^{-1}), \end{aligned}$$

and hence Eq. 2.4.

#### Proof of Theorem 2.3 for $d= 2$

 Take $d=2, k \ge 2$. Let $\beta \in \mathbb {R}$ and define $(r_t)_{t > 0}$ by Eq. 5.5, so that $t \pi f_0 r_t^2 - \log t +(3-2k) \log \log t = \beta$ for *t* large.

First suppose $k=2$. Set $v_{t}:=1+\frac{\log \log t}{\log t}$. By Eq. 4.2 and Proposition 5.3,

$$\begin{aligned} \gamma _t(A) = t f_0 \mathbb {E}[|V_{t,r_t,k}|]= e^{-\beta } + \sigma _A e^{-\beta /2} (\pi ^{1/2}/4) \Big ( 1 + \frac{\log \log t}{2 \log t} \Big ) + O((\log t)^{-1}). \end{aligned}$$

Hence by Lemma 4.1,

$$\begin{aligned} \mathbb {P}[R'_{t,\tau t,k} \le r_t] = \exp \Big (- \tau v_{t} e^{-\beta } -\tau \sigma _A e^{-\beta /2} (\pi ^{1/2}/4) \Big (1 + \frac{\log \log t}{2 \log t}\Big ) \Big ) + O((\log t)^{-1}) , \end{aligned}$$

and hence Eq. 2.8. Also by Lemma 4.2, with $\tau _n= m(n)/n$,

$$\begin{aligned} \mathbb {P}[R_{n,m(n),k} \le r_n] \!=\! \exp \Big (- \tau _{n} v_{n} e^{-\beta } \!-\!\tau _n \sigma _A e^{-\beta /2} (\pi ^{1/2}/4) \Big (1 \!+\! \frac{\log \log n}{2 \log n} \Big ) \Big ) + O((\log n)^{-1}) , \end{aligned}$$

and hence Eq. 2.7.

Now suppose $k \ge 3$. By Eq. 4.2 and Proposition 5.3,

$$\begin{aligned} \gamma _t(A) = \frac{\sigma _A e^{-\beta /2} \pi ^{1/2}}{(k-1)!2^k} \Big (1 + \frac{(2k-3)^2\log \log t}{2 \log t}\Big ) + O((\log t)^{-1}). \end{aligned}$$

Hence by Lemma 4.1,

$$\begin{aligned} \mathbb {P}[R'_{t,\tau t,k} \le r_t] = \exp \Big (- \frac{\tau \sigma _A e^{-\beta /2} \pi ^{1/2}}{ (k-1)!2^k} \Big (1 + \frac{(2k-3)^2 \log \log t}{2 \log t}\Big ) \Big ) + O((\log t)^{-1}), \end{aligned}$$

and hence Eq. 2.10 for $d=2, k \ge 3$ (note that $c_{2,k} = (\pi ^{1/2}) / ((k-1)!2^k)$ by Eq. 2.6). Also by Lemma 4.2, setting $\tau _n:= m(n)/n$, we have

$$\begin{aligned} \mathbb {P}[R_{n,m(n),k} \le r_n] = \exp \Big ( -\frac{\tau _n \sigma _A e^{-\beta /2} \pi ^{1/2}}{ (k-1)!2^k} \Big (1 + \frac{(2k-3)^2 \log \log n}{2 \log n} \Big ) \Big ) + O((\log n)^{-1}) , \end{aligned}$$

and hence Eq. 2.9 for $d=2, k \ge 3$.$\square$

#### Proof of Theorem 2.3 for $d\ge 3$

 Assume $d \ge 3, k \ge 1$. Let $\beta >0$ and let $(r_t)_{t >0}$ satisfy Eq. 5.5, so $t f_0 \theta r_t^d = (2-2/d) \log t +(2k-4 +2/d) \log \log t + \beta$ for *t* large. Then by Proposition 5.1, given $\varepsilon >0$, we have Eq. 5.7, and hence

$$\begin{aligned} \gamma _t(A) = t f_0 \mathbb {E}[|V_{t,r_t,k}|]&= e^{-\beta /2} c_{d,k} \sigma _A \Big (1 + \frac{ (k-2 + 1/d)^2 \log \log t}{(1-1/d)\log t} \\&+ \frac{ (k-2 + 1/d) \beta + 4k -4}{(2-2/d) \log t} \Big ) + O\big ( (\log t)^{\varepsilon -2} \big ). \end{aligned}$$

Hence by Lemma 4.1 we have

5.15 $$\begin{aligned} \mathbb {P}[R'_{t,\tau t,k} \le r_t]&= \exp \Big ( - \tau e^{-\beta /2} c_{d,k} \sigma _A \Big (1 + \frac{ (k-2 + 1/d)^2 \log \log t}{(1-1/d) \log t} \nonumber \\&+ \frac{ (k-2 + 1/d) \beta + 4k -4}{(2-2/d) \log t} \Big ) \Big ) + O\big ((\log t)^{\varepsilon -2} \big ), \end{aligned}$$

which gives us Eq. 2.10, and by Lemma 4.2, setting $\tau _n = m(n)/n$ we have

5.16 $$\begin{aligned} \mathbb {P}[R_{n,m,k} \le r_t]&= \exp \Big ( - \tau _n e^{-\beta /2} c_{d,k} \sigma _A \Big (1 + \frac{ (k-2 + 1/d)^2 \log \log n}{(1-1/d) \log n} \nonumber \\&+ \frac{ (k-2 + 1/d) \beta + 4k -4}{(2-2/d) \log n} \Big ) \Big ) + O\big ( (\log n)^{\varepsilon -2} \big ), \end{aligned}$$

giving us Eq. 2.9.$\square$

## Simulation Results and Discussion

**Fig. 5:**
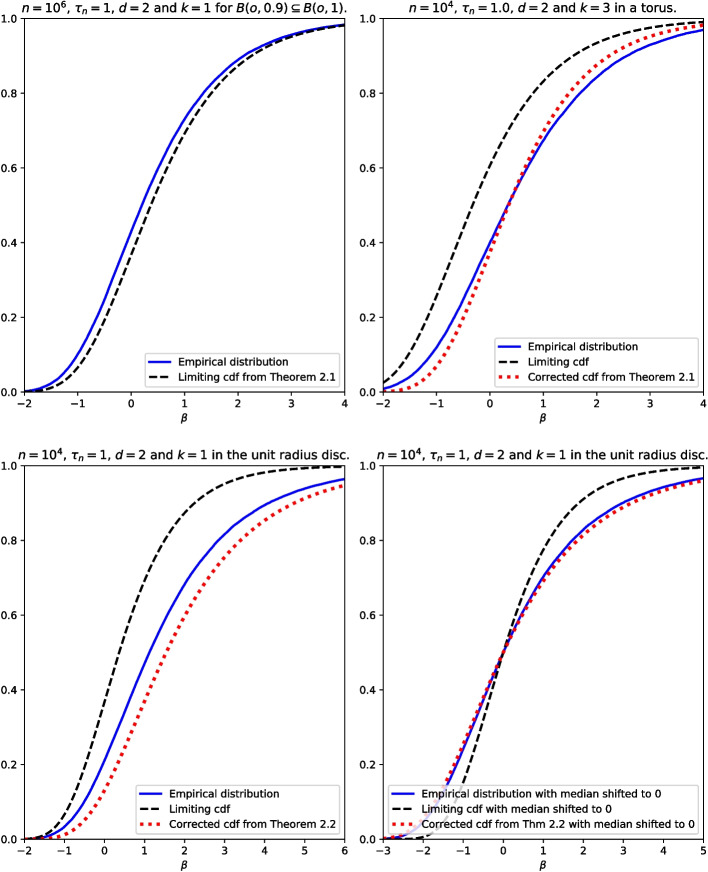
The empirical distributions of $n \theta _d f_0 R_{n,m(n),k}^d - c_1 \log n - c_2 \log \log n$ obtained from computer simulations in the settings of Theorems 2.1, 2.2 and 2.3, plotted on the same axes as the limiting distributions. See Section 6 for discussion of the simulation results

**Fig. 6:**
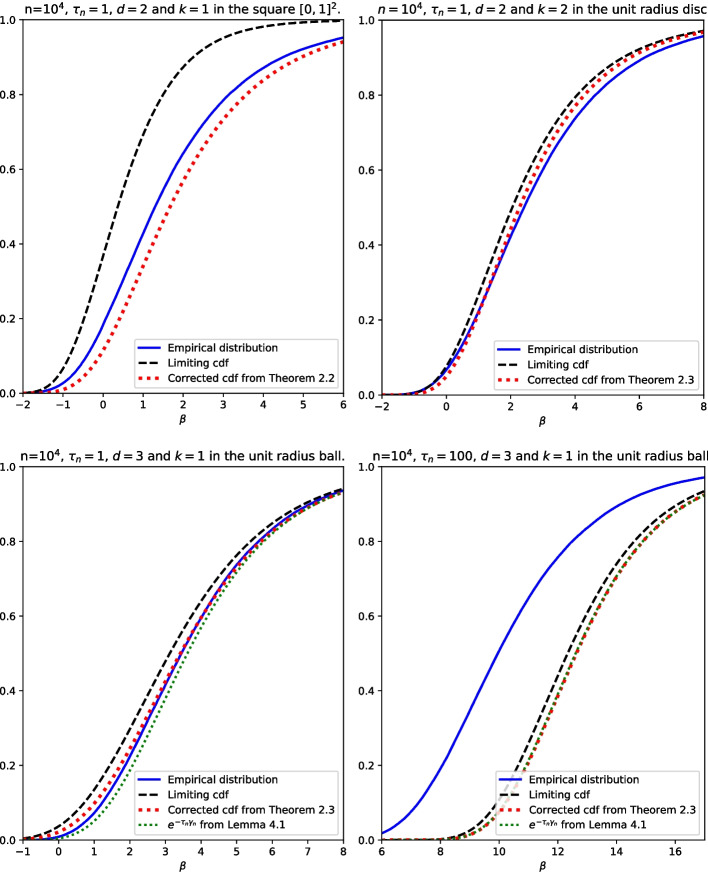
The continuation of Fig. 5, with results from simulations in several more settings

We were able to write computer simulations which sample from the distribution of $R_{n,m(n),k}$ using a very simple algorithm: sample $n + m(n)$ independent points $X_1, \dots , X_n, Y_1, \dots , Y_{m(n)}$. For each $j \in \{1, \dots , m(n)\}$ let $d_j^{(k)}$ be the Euclidean distance between $Y_j$ and its *k*th-nearest point in $\mathcal {X}_n = \{ X_1, \cdots , X_n \}$. Then $R_{n,m(n),k} = \max _{j \le m(n)} d_j^{(k)}$.

In Figs. 5 and 6, we present the results from simulations of many of the settings for which we have proved limit theorems. In each of the eight plots, the blue curve is an estimate of the cumulative distribution function of the quantity of the form $n \theta _d f_0 R_{n,m(n),k}^d - c_1 \log n - c_2 \log \log n$ for which we have obtained weak laws. These distributions were estimated by sampling several tens of thousands of times from the distribution of $R_{n,m(n),k}$ and plotting the resulting empirical distribution. The black dashed curves are the corresponding limiting distributions as $n \rightarrow \infty$, from Theorems 2.1, 2.2 and 2.3. The red dotted curves are the corresponding “corrected” distributions, i.e. the explicit distributions which occur on the right-hand side of the expressions in our limit theorems, neglecting only the errors of order $(\log n)^{-1}$.

The top row in Fig. 5 shows two cases covered by Theorem 2.1. The top-left diagram is for $B = B(o,0.9)$, $A = B(o,1)$, which meets condition A3. We have $d=2$ and $k=1$, so there is no “correction” to the limiting distribution. This is the only diagram in which we have taken *n* larger than $10^4$. When plotted for $n = 10^4$ (not pictured), the distance between the empirical distribution and the limiting distribution appears to be smaller than for $n = 10^6$, but the shapes of the curves are very different, indicating that there is still a boundary effect influencing the distribution of the two-sample coverage threshold.

The top-right diagram is for our results when points are placed on the 2-dimensional unit torus. As a remark following Theorem 2.1 states, the proof of that theorem would generalise to this setting, giving exactly the same result. We have simulated $R_{n,m(n),k}$ for $k=3$, which is a case covered by our Theorem 2.1 but not included in the results of Iyer and Yogeshwaran (2012).

Both diagrams on the bottom row of Fig. 5 are representations of the same simulation, with points placed inside the two-dimensional unit disc, which certainly has a smooth boundary. The inclusion of the explicit term of order $(\log n)^{-1/2}$ improves the accuracy of the estimated distribution considerably, as can be seen from the fact that the red dotted curve in the left diagram is much closer to the empirical distribution than the black dashed curve. In this $d=2$, $k=1$ setting the correction is of a larger order than the $O( \frac{\log \log n}{\log n} )$ terms in all of the other settings.

We remarked after stating Theorem 2.2 that $n \pi f_0 R_{n,m(n)}^2 - \mu (n \pi f_0 R_{n,m(n)}^2) \overset{\mathcal {D}}{\longrightarrow }{\textsf{Gu}}_{\log \log 2,1}$, where $\mu (\cdot )$ is the median and ${\textsf{Gu}}_{\log \log 2,1}$ is a Gumbel distribution with scale parameter 1 and median 0. To illustrate this, in the second diagram on the second row of Fig. 5 we have translated all of the curves from the first diagram so that they pass through (0, 1/2), i.e. so that they are the distributions of random variables with median 0. We can see that the corrected distribution is very close to the empirical distribution from the simulation, indicating that the *shape* of the corrected limiting distribution closely matches the actual distribution of $n \pi f_0 R_{n,m(n)}^2$ for finite *n*, but with an offset corresponding to the difference between $\log n$ and the median of $n \pi f_0 R_{n,m(n)}^2$.

In the setting of Theorem 2.2, the presence of a boundary has an effect on the distribution of $n \pi f_0 R_{n,m(n)}^2$ which disappears as $n \rightarrow \infty$, so is not reflected in the limit. Broadly speaking, the terms involving $e^{-\beta }$ come from the interior, and terms involving $e^{-\beta /2}$ come from the boundary. Our correction term corrects the shape of the distribution to account for these boundary effects.

The blue curve in the left-hand diagram was translated by the *sample* median in order to pass through (0, 1/2) in the right-hand diagram. However, for applications of these limit theorems to real data, it is unlikely that tens of thousands of independent samples of $R_{n,m}$ will be available to estimate the median of the distribution.

Theorem 2.2 covers two cases for $d=2$, $k=1$: when *A* has a smooth boundary, and when *A* is a polygon. The first diagram in Fig. 6 is in this latter case, with $A = [0,1]^2$. If we compare this diagram with the bottom-left diagram of Fig. 5, which is also for $d=2$, $k=1$ but with $A = B(o,1)$, all of the same qualitative features can be observed: a fairly large gap between the empirical distribution and the limit, a large improvement due to the correction, and an “overshoot” so the corrected distribution approximates the empirical distribution from the right-hand side while the limiting distribution is to the left.

This indicates that the behaviour of the two-sample coverage threshold (at least in two dimensions) is not strongly affected by the presence of “corners” on the boundary of *A*. It is likely that in higher dimensions, the limiting behaviour of $R_{n,m(n)}$ when *A* is a polytope would be different from the behaviour when *A* has a smooth boundary, as was observed for the *coverage threshold* in Penrose (2023).

The top-right diagram in Fig. 6 is for $d=2$, $k=2$ with points inside the unit disc, which is the setting of the first limit result in Theorem 2.3. The $d=2$, $k=2$ case is unique in that the limiting distribution has two terms, corresponding to the boundary and interior. In the other settings the limiting distribution for the position of the point in $\mathcal {Y}_j$ which is last to be *k*-covered as the discs expand is either distributed according to Lebesgue measure on *A*, or according to a distribution supported on $\partial A$. However, the existence of both terms in the limit in Eq. 2.7 indicates that for $d=k=2$, the “hardest point to *k*-cover” has a mixed distribution: the sum of a measure supported on the interior of *A* with a measure supported on $\partial A$.

The bottom row of Fig. 6 contains the distributions from two simulations with $d=3$ and $k=1$ inside the unit ball. In the left diagram we have taken $\tau = 1$, and the corrected limit approximates the empirical distribution well. In the right diagram we have taken $\tau = 100$. The empirical distribution is extremely far from the limiting distribution, and the correction term has the wrong sign, so the corrected limit is an even worse approximation to the empirical distribution than the uncorrected limit is.

The fact that the empirical distribution is far to the *left* of the limit (i.e. that $R_{n,m(n),k}^d$ is generally smaller than the limit would predict) when $\tau$ is large is rather surprising. If we consider $\tau _n \uparrow \infty$ sufficiently fast as $n \rightarrow \infty$, then $R_{n,m(n),k}$ should approximate the *coverage threshold* considered in Penrose (2023). As we remarked after the statement of Theorem 2.3, the coverage threshold is generally much larger than our $R_{n,m(n),k}$. In the case $d=3$, $k=1$, the coefficient of $\log \log n$ in the weak law for the coverage threshold corresponding to Theorem 2.3 is larger, and so we might expect that if $\tau$ is large than the empirical distribution for $n \theta _d f_0 R_{n,m(n),k}^d - (2-2/d)\log n - (2k-4+2/d)\log \log n$ in Fig. 6 would be far to the right of the limiting distribution.

To explain the surprising fact that it is instead far to the left of the limit, we should examine Lemma 4.1. In the “Poissonized” setting of that Lemma, given the configuration of “transmitters” $\mathcal {X}_t$, the conditional probability $\mathbb {P}[R_{t,\tau t,k}' \le r_t | \mathcal {X}_t]$ is the probability that no point from $\mathcal {Y}_{\tau t}$ lies in the vacant region $V_{t,\tau t,k}$. The lemma shows that when we replace the marginal probability ${\mathbb P}[R_{t,\tau t,k}' \le r_t]$ with the probability that no point from $\mathcal {Y}_{\tau t}$ lies in a region of Lebesgue measure $\mathbb {E}V_{t,\tau t, k}$, the error induced is $O( (\log t)^{1-d} )$. However, this is for fixed $\tau$. It can be seen from the proof that the error is proportional to $\tau ^2 (\log t)^{1-d}$, which is not negligible unless *t* is very large compared to $\tau$.

To see why the corrected limiting cdf is *below* the empirical cdf, let $f(x):= e^{-\tau x}$. In Lemma 4.1, if $\Gamma _t:= (t / |B|) |V_{t,r_t,k} \cap B |$, then ${\mathbb P}[ R_{t,\tau t,k}'(B) \le r_t ] = \mathbb {E}[f( \Gamma )]$, while $e^{-\tau \gamma _t(B)} = f( \mathbb {E}[\Gamma ] )$. Hence by Jensen’s inequality, $e^{-\tau \gamma _t}$ can only ever be an *underestimate* for ${\mathbb P}[ R_{t,\tau t,k}'(B) \le r_t ]$, with an error proportional to $\tau ^2$. All of our corrected expressions in Theorem 2.3 are approximations of $e^{-\tau \gamma _t}$.

If we think of $\mathcal {X}_n$ as a set of transmitters and $\mathcal {Y}_{m(n)}$ as a set of receivers, then for most applications we would expect $\tau _n$ to be large. It should be possible to improve the estimate in this case by computing the leading-order error terms in Lemma 4.1, using moments of $t|V_{t,\tau t,k}|$ or otherwise.

## Data Availability

The code for the simulations discussed in Section 6 is available at https://github.com/frankiehiggs/CovXY and the samples generated by that code are available at https://researchdata.bath.ac.uk/id/eprint/1359.
